# Structure and dynamics of a mycobacterial type VII secretion system

**DOI:** 10.1038/s41586-021-03517-z

**Published:** 2021-05-12

**Authors:** Catalin M. Bunduc, Dirk Fahrenkamp, Jiri Wald, Roy Ummels, Wilbert Bitter, Edith N. G. Houben, Thomas C. Marlovits

**Affiliations:** 1 Centre for Structural Systems Biology, Hamburg, Germany; 2 Institute of Structural and Systems Biology, University Medical Centre Hamburg–Eppendorf, Hamburg, Germany; 3 Deutsches Elektron Synchrotron DESY, Hamburg, Germany; 4 Molecular Microbiology Section, Amsterdam Institute of Molecular and Life Sciences, Vrije Universiteit Amsterdam, Amsterdam, The Netherlands; 5 Department of Medical Microbiology and Infection Control, Amsterdam Infection and Immunity Institute, Amsterdam UMC, Amsterdam, The Netherlands

**Keywords:** Membrane proteins, Bacterial pathogenesis, Bacterial secretion, Bacterial structural biology, Cryoelectron microscopy

## Abstract

*Mycobacterium tuberculosis* is the cause of one of the most important infectious diseases in humans, which leads to 1.4 million deaths every year^1^. Specialized protein transport systems—known as type VII secretion systems (T7SSs)—are central to the virulence of this pathogen, and are also crucial for nutrient and metabolite transport across the mycobacterial cell envelope^2,3^. Here we present the structure of an intact T7SS inner-membrane complex of *M. tuberculosis*. We show how the 2.32-MDa ESX-5 assembly, which contains 165 transmembrane helices, is restructured and stabilized as a trimer of dimers by the MycP_5_ protease. A trimer of MycP_5_ caps a central periplasmic dome-like chamber that is formed by three EccB_5_ dimers, with the proteolytic sites of MycP_5_ facing towards the cavity. This chamber suggests a central secretion and processing conduit. Complexes without MycP_5_ show disruption of the EccB_5_ periplasmic assembly and increased flexibility, which highlights the importance of MycP_5_ for complex integrity. Beneath the EccB_5_–MycP_5_ chamber, dimers of the EccC_5_ ATPase assemble into three bundles of four transmembrane helices each, which together seal the potential central secretion channel. Individual cytoplasmic EccC_5_ domains adopt two distinctive conformations that probably reflect different secretion states. Our work suggests a previously undescribed mechanism of protein transport and provides a structural scaffold to aid in the development of drugs against this major human pathogen.

## Abstract (web summary)

A cryo-electron microscopy structure of the inner membrane complex of the ESX-5 type VII secretion system of *Mycobacterium tuberculosis* reveals an important role of interactions with MycP_5_ protease for complex integrity.

## Main

*Mycobacterium tuberculosis* encodes five homologous, but functionally distinct, T7SSs that are designated ESX-1 to ESX-5. These systems translocate a number of effector proteins across the unique and impermeable diderm cell envelope^2,3^. Because of their importance for mycobacterial physiology and virulence, T7SSs are considered to be promising targets for the development of drugs for the treatment or prevention of tuberculosis^4^. Although T7SSs have previously been shown to form hexameric complexes^5^, high-resolution structural information exists only for part of the T7SS—a dimeric ESX-3 subcomplex from the nonpathogenic species *Mycobacterium smegmatis*^6,7^. Here we reconstituted the ESX-5 T7SS of *M. tuberculosis* H37Rv in *M. smegmatis* to obtain a structural view of the entire T7SS membrane complex from this human pathogen.

## Architecture and stoichiometry

**Extended Data Fig. 1:**
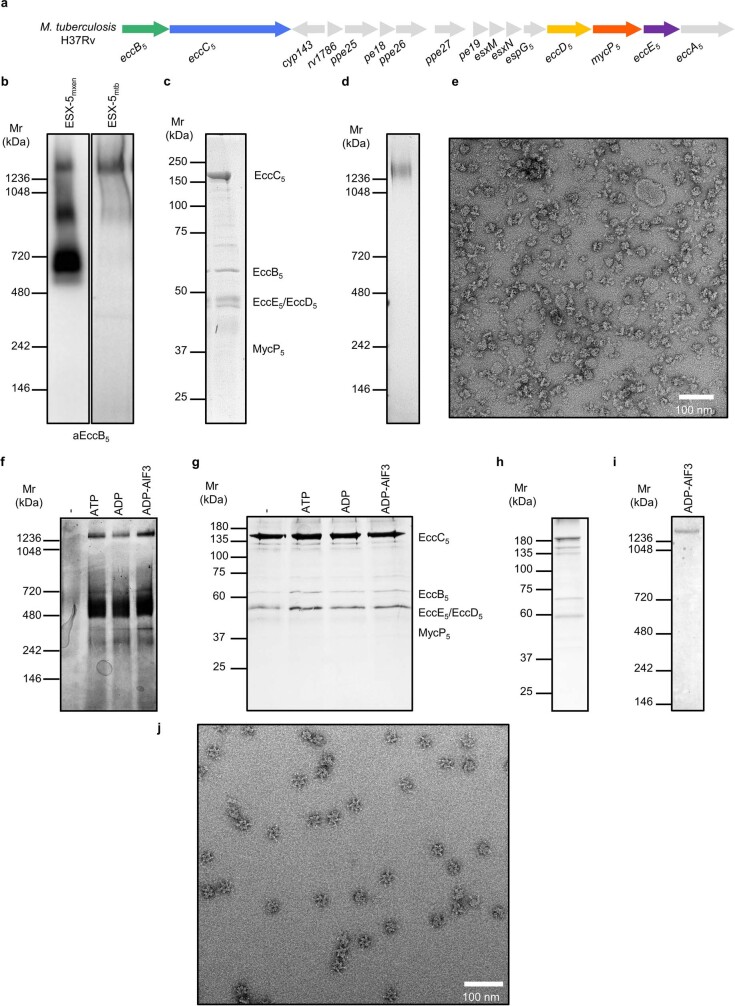
Purification of the *M. tuberculosis* ESX-5 membrane complex. **a**, Genetic organization of the *esx-5* locus of *M. tuberculosis* H37Rv, which has been cloned and expressed in *M. smegmatis* MC^2^155. **b**, BN-PAGE and western blot analysis using an anti-EccB_5_ antibody of DDM-solubilized membranes from *M. smegmatis* MC^2^155 expressing *M. xenopi* or *M. tuberculosis* ESX-5 (ESX-5_mxen_ or ESX-5_mtb_, respectively). Experiment was reproduced three times. **c**, **d**, Coomassie-stained SDS–PAGE (**c**) and BN-PAGE (**d**) of Strep- and SEC-purified ESX-5_mtb_ membrane complexes. **e**, Negative-stain electron microscopy analysis of ESX-5_mtb_ membrane complexes shown in **c** and **d**. Experiments in **c**–**e** were replicated three times. **f**, BN-PAGE and Coomassie staining of Strep-purified ESX-5_mtb_ complexes without nucleotides (−) or in the presence of nucleotides ATP, ADP or the transition-state analogue ADP–AlF_3_. Upon purification, in the presence of either nucleotide, the higher-molecular-weight species of the membrane complex becomes more prominent. **g**, SDS–PAGE and Coomassie staining of the same samples as in **f**, showing a similar SDS–PAGE protein pattern between the four conditions. Experiment in **f**, **g** was performed three times. **h**, **i**, Coomassie-stained SDS–PAGE (**h**) and BN-PAGE (**i**) of Strep- and SEC-purified ESX-5_mtb_ membrane complexes in the presence of ADP–AlF_3_. **j**, Negative-stain electron microscopy of the same sample as in **h**, **i**, showing improved sample homogeneity as compared to purifications in the absence of nucleotides, as shown in **e**. Experiment shown in **h**–**j** was performed twice.

The *M. tuberculosis* ESX-5 system showed robust expression in *M. smegmatis* and correct assembly of the membrane complex (Extended Data Fig. 1a, b). Purification of the *M. tuberculosis* ESX-5 membrane complex (using a C-terminal Strep tag on EccC_5_ and mild solubilization conditions) resulted in copurification of the conserved MycP_5_ protease (Extended Data Fig. 1c–e). To our knowledge, MycP (also known as mycosin) is absent in all previously reported T7SS structures^5–7^—although MycP is known to be essential for T7SS function and complex stability^8^. The addition of nucleotides and MgCl_2_ improved sample homogeneity, as judged by a more-distinct high molecular weight complex on native-PAGE, size exclusion chromatography and subsequent negative-stain electron microscopy analysis (Extended Data Fig. 1f–j).

**Fig. 1:**
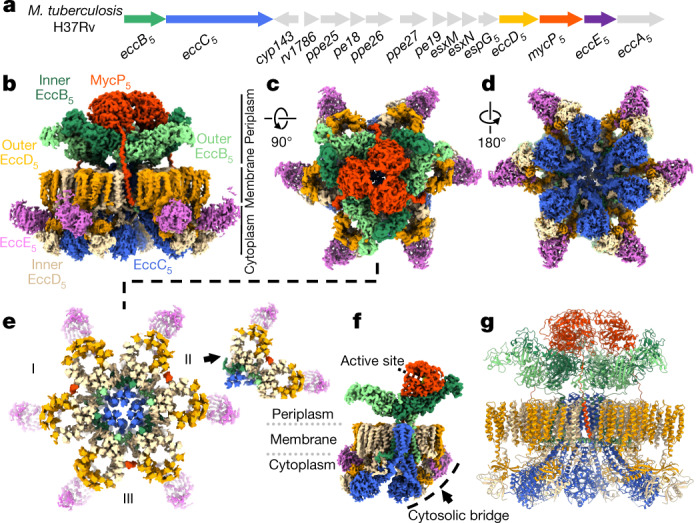
Cryo-EM structure of the intact ESX-5 inner-membrane complex of *M. tuberculosis*. **a**, Genetic organization of the *esx-5* locus of *M. tuberculosis* H37Rv, which was cloned and expressed in *M. smegmatis* MC^2^155. **b**–**e**, Cryo-EM density of the intact ESX-5 inner-membrane complex of *M. tuberculosis*, zoned and coloured for every individual component. Components are inner EccB_5_ (dark green), outer EccB_5_ (light green), EccC_5_ (blue), inner EccD_5_ (beige), outer EccD_5_ (orange), EccE_5_ (purple) and MycP_5_ (red). The full complex is 28.5 nm in width and 20 nm in height, and has an absolute stoichiometry of 6:6:12:6:3 for EccB_5_:EccC_5_:EccD_5_:EccE_5_:MycP_5_. **b**–**e**, Side (**b**), top (**c**) and bottom (**d**) views and a top cross-section (**e**) of the complex at the membrane level, highlighting the arrangement of the 165-TMH region. Inset, top cross-section of an extracted dimeric unit. **f**, Single dimer viewed from the centre of the intact complex, highlighting the central EccC_5_ TMH bundle and the position of MycP_5_ with its active site directed towards the inside of the periplasmic cavity. **g**, Ribbon model of the *M. tuberculosis* ESX-5 assembly.

**Extended Data Fig. 2:**
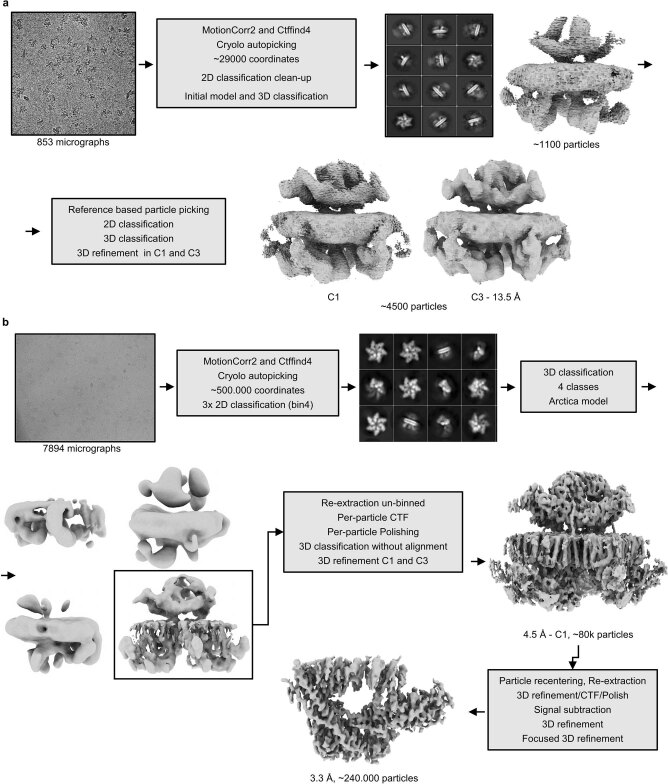
Cryo-EM data collection and single-particle reconstruction procedure. **a, b**, This figure relates to the initial Talos-Arctica-collected dataset (**a**) and the first higher-resolution Titan-Krios-collected dataset (**b**).

**Extended Data Fig. 3:**
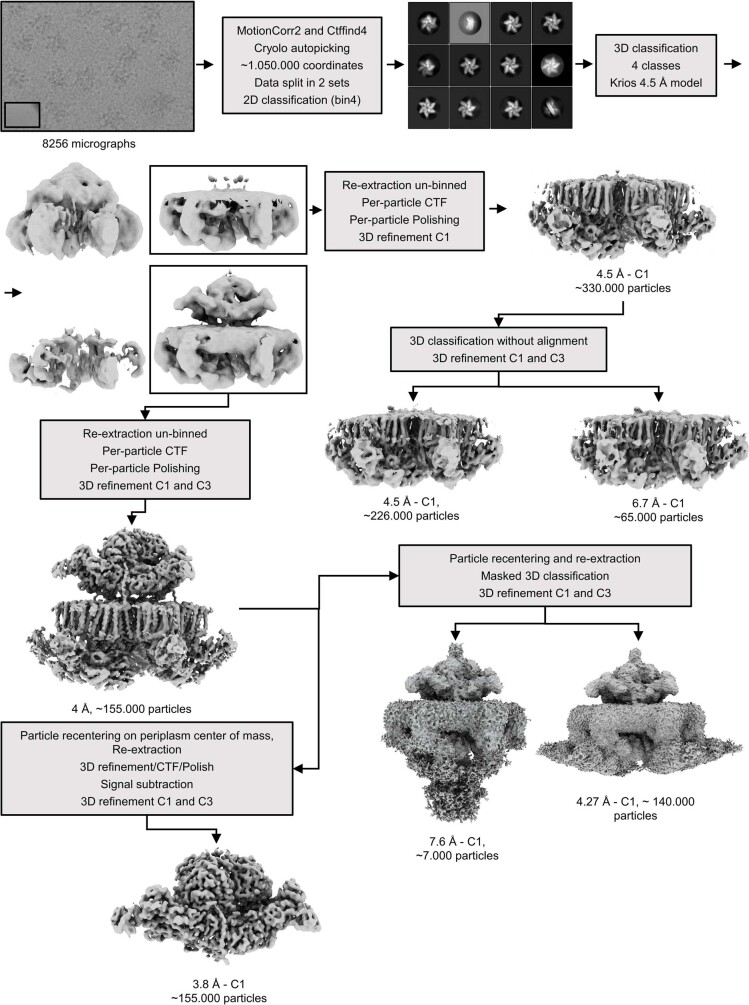
Cryo-EM data collection and single-particle reconstruction procedure. This figure relates to the second high-resolution Titan Krios collected dataset.

**Extended Data Fig. 4:**
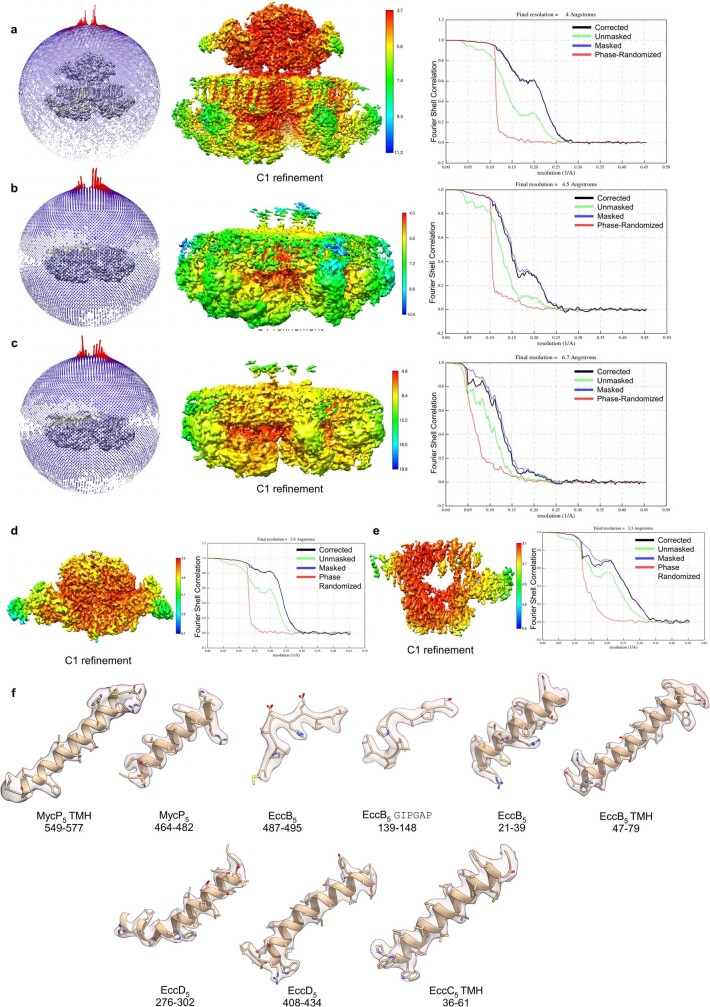
Single-particle reconstructions of the ESX-5_mtb_ membrane complex. **a–c**, Angular distribution plots, local-resolution estimations and Fourier shell correlation (FSC) plots of the C1 reconstruction of the entire MycP_5_-bound ESX-5_mtb_ membrane complex (**a**), and C1 reconstructions of the two heterogeneous MycP_5_-unbound ESX-5_mtb_ membrane complexes (**b**, **c**). **d**, **e**, Local-resolution estimation and FSC plot for the C1-refined periplasmic map (**d**) and the map of the cytosolic bridge (**e**). **f**, Examples of cryo-EM densities and corresponding models.

**Extended Data Fig. 5:**
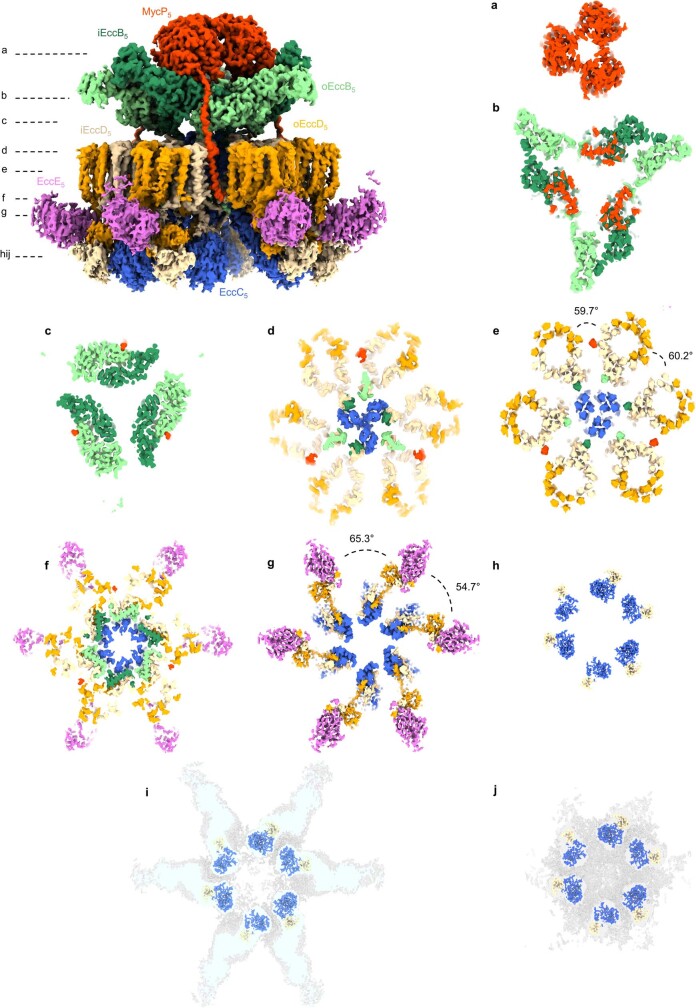
Top cross-sections through the intact ESX-5_mtb_ membrane complex. **a**, MycP_5_ trimer top view, highlighting the pore formed at the periplasmic side. **b**, Section through the periplasmic assembly at the EccB_5_–MycP_5_ interface, showing the position of the protease domain sitting on top of inner EccB_5_. **c**, Section through the periplasmic assembly at the EccB_5_ dimer interface level, highlighting the MycP_5_ linker connection to the TMH. **d**, Top view of the six membrane protomers with the closed EccC_5_ TMH pyramid at the centre. **e**, Top cross-section through the six membrane protomers, highlighting 153 of the 165 TMHs. At the central area towards the cytosol, the three-EccC_5_ TMH pyramid opens up in a manner similar to an iris. MycP_5_, the protease domain of which interacts with the protomer containing inner EccB_5_, interacts with the outer EccD_5_ barrel of the adjacent protomer at the membrane level. At the membrane level, the angle between protomers within a dimer and between adjacent protomers of different dimers differs by only 0.5°. **f**, Top section displaying the region below the inner leaflet of the inner membrane, highlighting a further opening of the EccC_5_ gated pore and the lower part of the EccB_5_ basket, formed by EccB_5_ N termini. **g**, At the cytosolic level, the angle between protomers differs to that at the membrane level. As such, the angle between protomers within a dimer grows to 65.3°, while the angle between adjacent protomers of different dimers decreases to 54.7°. The change in angles between the membrane and cytosolic regions of protomers is caused by MycP_5_ binding, which induces a slight tilting to the protomers that it binds via inner EccD_5_. **h**, Section through the lower region of the cytosolic bridge, containing the DUF domain of EccC_5_ and the cytosolic domain of inner EccD_5_. **i**, Same view as in **h**, but overlaid with the EccC_5_ extended state, highlighting the radial extension of the EccC_5_ NBD1, NBD2 and NBD3 almost parallel to the inner membrane. **j**, Same view as in **h**, but then overlaid with the EccC_5_ contracted stated.

Cryo-electron microscopy (cryo-EM) analysis of the *M. tuberculosis* ESX-5 complex purified in the presence of ADP–AlF_3_ showed clear hexameric particles (Extended Data Figs. 2b, 3). We performed an ab initio reconstruction without symmetry enforcement that yielded an average resolution of approximately 4 Å (Extended Data Fig. 4), which improved to an overall resolution of approximately 3.5 Å after further data processing; this allowed us to build around 78% of the stable complex de novo (Supplementary Tables 1, 2). The intact machinery comprises EccB_5_, EccC_5_, EccD_5_, EccE_5_ and MycP_5_ with a 6:6:12:6:3 stoichiometry (Fig. 1b, c, d, Supplementary Video 1) resulting in a 2.32-MDa complex that is anchored in the inner membrane through 165 transmembrane helices (TMHs) (Fig. 1e). The membrane assembly is best described as a trimer of dimers, in which each dimer comprises a single copy of MycP_5_ and two protomers each of one copy of EccB_5_, EccC_5_, EccE_5_ and two copies of EccD_5_ (Fig. 1f). The overall fold and stoichiometry of a dimeric building block of *M. tuberculosis* ESX-5 is similar to that of a dimer of ESX-3 from *M. smegmatis*^6,7^, albeit with notable differences on the periplasmic side and the angle between protomers. In the intact ESX-5 complex, the angle between protomers at the membrane level differs by about 0.5°, from 59.7° between protomers of one dimer to 60.2° between protomers of adjacent dimers. However, at the cytosolic level these angles differ by more than 10° (from 65.3° to 54.7°) (Extended Data Fig. 5). By contrast, the individual ESX-3 dimer displayed an overall angle of 72° between protomers^6,7^.

## Structural rearrangements of periplasmic domains

**Fig. 2:**
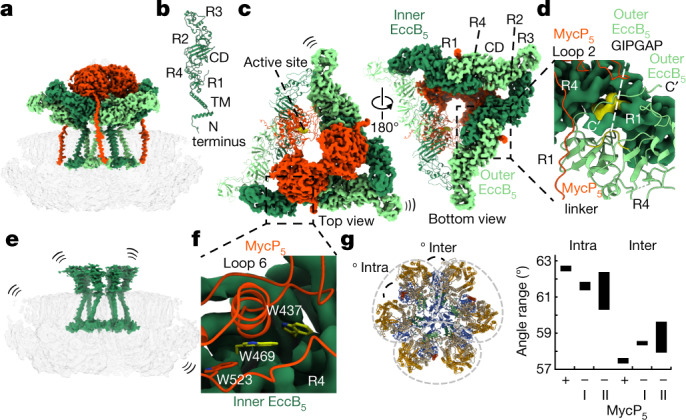
MycP_5_ drives EccB_5_ hexamerization and stabilization of the membrane complex. **a**, Transparent assembly of intact *M. tuberculosis* ESX-5, with EccB_5_ and MycP_5_ coloured as in Fig. 1. **b**, Complete structure of monomeric *M. tuberculosis* EccB_5_, highlighting its overall fold and domains. **c**, Top and bottom view of the EccB_5_–MycP_5_ periplasmic assembly with one unit (EccB_5_ dimer and MycP_5_ monomer) as ribbon model, highlighting the active site of MycP_5_ in yellow. CD, central domain; TM, TMH. **d**, EccB_5_ dimerization site, highlighting the C-terminus of outer EccB_5_ that is wrapped around the R1 and R4 domains of the adjacent inner EccB_5_ monomer, the conserved GIPGAP motif of EccB_5_ (in yellow) and the interactions of the EccB_5_ dimer with loop 2 and the linker connection of MycP_5_. **e**, Transparent map of *M. tuberculosis* ESX-5 without copurified MycP_5_, with EccB_5_ highlighted in dark green. The high flexibility of EccB_5_ and the overall heterogeneity of the membrane complex in the absence of MycP_5_ is indicated by curved lines. **f**, EccB_5_–MycP_5_ interaction surface, highlighting the three buried tryptophans. **g**, Angle variation range between protomers of the MycP_5_-bound (+) and two unbound (−) states (I and II). Intra, between two protomers within a dimer: 62.7°, 62.4° and 62.4° (MycP_5_-bound); 61.8°, 61.3° and 61.5° (unbound, I); 61.4°, 62.3° and 60.2° (unbound, II). Inter, between two protomers of adjacent dimers: 57.5°, 57.3° and 57.5° (MycP_5_-bound); 58.5°, 58.3° and 58.3° (unbound, I); and 57.9°, 58.3° and 59.6° (unbound, II).

**Extended Data Fig. 6:**
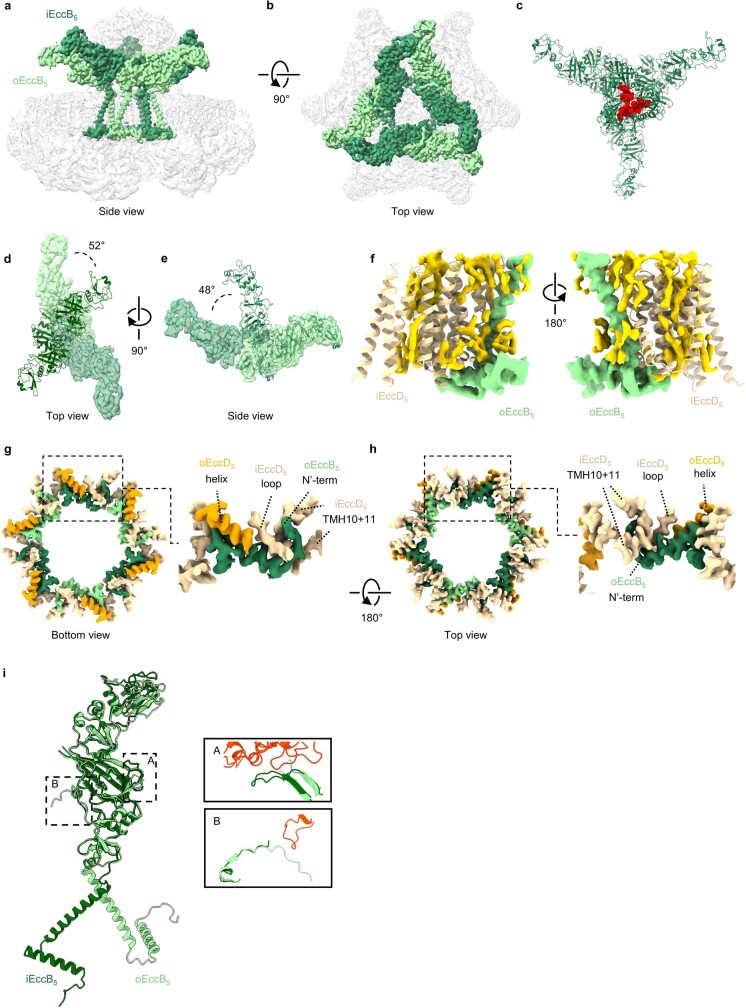
Hexameric EccB_5_ adopts a triangular conformation in the periplasm. **a**, **b**, Side (**a**) and top **(b)** view of an intact ESX-5_mtb_ assembly in which inner EccB_5_ and outer EccB_5_ are coloured as in Fig. 1 and the rest of the components are transparent. **c**, A V-shaped EccB_3_ dimer (PDB 6SGY) was fitted into the *M. smegmatis* ESX-3 dimer cryo-EM density (EMDB EMD-20820) together with the corresponding dimeric ESX-3 model for the membrane and cytosolic domains (PDB 6UMM) using the Chimera fit in map tool. This composite dimer model was subsequently trimerized, on the basis of our full ESX-5 map reconstructions. The clashing of EccB_3_ periplasmic domains between the dimers, towards the central area, in this hybrid model are highlighted in red. **d**, Upon MycP_5_ binding to the assembly, the periplasmic EccB_5_ dimer is rotated by 52°, avoiding the clashes observed in **c**. Angles were measured by aligning the hybrid model and the ESX-5_mtb_ model at the membrane level. Subsequently, centres of mass were defined for the combined R1 domains of each EccB_3_ and EccB_5_ dimer (at the base of the dimer) and for every R2 and R3 EccB monomer (toward the tips of the EccB dimer). Planes defined by these three points were generated for both EccB_3_ and EccB_5_ dimers and angles were measured between these two planes. EccB_3_ dimer is shown as a ribbon model and EccB_5_ model is shown as zoned density. **e**, Compared to the V-shaped EccB_3_ dimer (ribbon model), the angle between the two EccB_5_ monomers (zoned density) grows by 48° upon MycP_5_ binding. EccB dimer angles were calculated by measuring the angle between the centres of mass of the R2 and R3 domains of each EccB protomer in relation to the centre of mass of both R1 domains. **f**, Side views of the TMH region of inner EccD_5_, depicted as a ribbon model, and the TMH and N terminus of an interacting outer EccB_5_, depicted as zoned density. An array of lipids found in the EccD_5_ barrel (but also surrounding this inner EccD_5_–EccB_5_ interaction site) are depicted in gold. **g**, Bottom view of the lower cytosolic area of the EccB_5_ basket, formed by EccB_5_ N termini (residues 10–48) and depicted with the interacting pocket formed by TMH10, TMH11 and TMH8 (not shown for clarity) of inner EccD_5_ of the adjacent protomer. The EccB_5_ N terminus is also buttressed in this position by a short helix (residues 119–130) of outer EccD_5_, which connects outer EccD_5_ TMHs with its cytosolic domain, and also by part of the inner EccD_5_ loop (residues 307–315) that subsequently folds along the stalk and DUF domain of EccC_5_. **h**, Same map as in **g**, but viewed from the top. **k**, Superposition of inner EccB_5_ and outer EccB_5_, highlighting conformational differences between the two, which are the result of the interaction with MycP_5_.

The periplasmic assembly of the ESX-5 membrane complex of *M. tuberculosis* is formed by three EccB_5_ dimers and three MycP_5_ proteases. The EccB_5_ dimers assemble in a triangle, which forms a central cavity (Fig. 2c). Within an EccB_5_ dimer, two slightly different conformations (that is, inner and outer) can be distinguished between monomers, depending on their position. EccB_5_ dimerization is mediated mainly through the R1 and R4 repeat domains and is further stabilized by the EccB_5_ C termini, which wrap around their interacting EccB_5_ partner to form intermolecular hydrophobic contacts with its R1 domain (Fig. 2d). The GIPGAP motif—which is a highly conserved region in EccB homologues—is central to these interactions (Fig. 2d). Compared to the EccB_3_ dimer from the ESX-3 subassembly^6,7^, the three EccB_5_ dimers are rotated by about 52° with respect to their corresponding EccC_5_–EccD_5_–EccE_5_ membrane dimers, which indicates that large conformational rearrangements are required during maturation into the fully assembled hexamer (Extended Data Fig. 6d). To form the triangle-shaped assembly, the inner EccB_5_ engages the outer EccB_5_ of the adjacent dimer by packing its R3 domain against the α-helices α5 and α8 of domains R2 and R3, respectively, which results in an asymmetric EccB_5_–tip arrangement (Fig. 2c). Consequently, domain R3 of the outer EccB_5_ does not form any interactions at its tip extremity and thus displays higher flexibility, consistent with previous observations^9^.

## Periplasmic MycP_5_–EccB_5_ assembly

**Extended Data Fig. 7:**
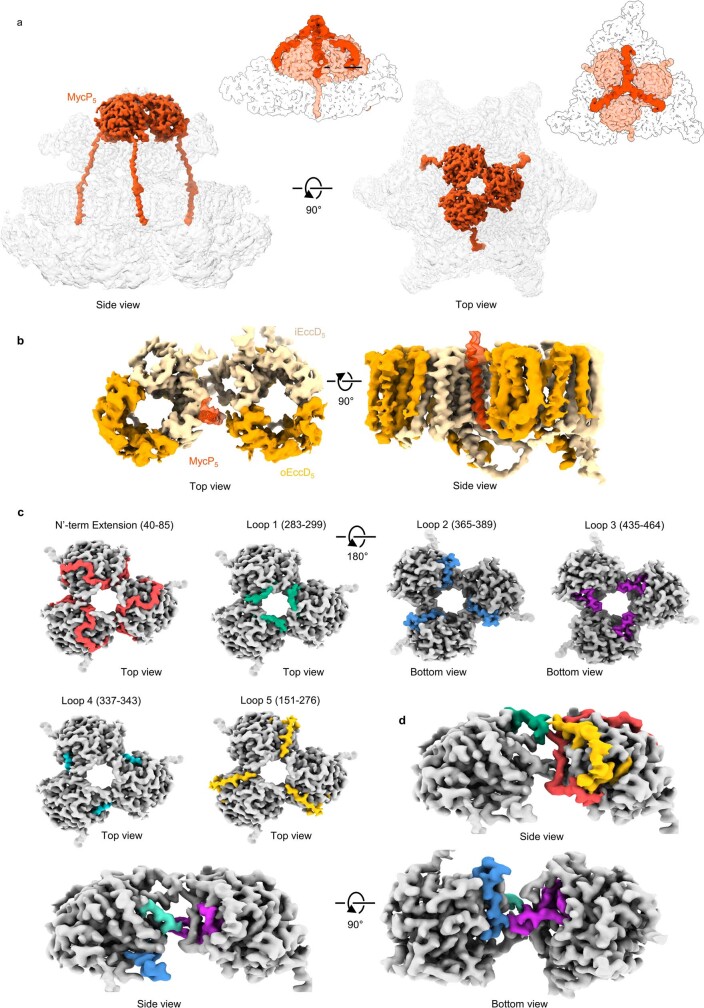
MycP_5_ caps a periplasmic cavity with its active site directed towards the lumen. **a**, Side and top view of an intact ESX-5_mtb_ assembly with MycP_5_ coloured as in Fig. 1 and the rest of the components transparent. Insets, side and top views of the periplasmic assembly with EccB_5_ in white, the MycP_5_ density shown in transparent red and loop 5 of MycP_5_ depicted in solid red at a higher threshold, to highlight it capping the periplasmic pore. Loop 5 folds along the protease domain, towards the pore formed by the MycP_5_ trimer. At higher thresholds, loop 5 caps this pore. **b**, Top and side view of a dimer of EccD_5_ barrels, of which one barrel (left) binds via inner EccD_5_ to the MycP_5_ TMH. **c**, Top or bottom view of MycP_5_ trimers depicted in grey with the loops that are involved in MycP_5_–MycP_5_ interactions depicted in different colours. **d**, Side and bottom views showing the MycP_5_–MycP_5_ interactions mediated by the same domains depicted in the same colours as in **e**.

Our periplasmic *M. tuberculosis* ESX-5 map shows three MycP_5_ proteases that form a dome-like structure, which cap the periplasmic central cavity (Fig. 2a, c, Supplementary Video 2). Interactions between EccB_5_ and MycP_5_ are mediated mainly by the MycP_5_ protease domain and a composite interface that is generated by the R4 domain and loop 6 (residues Thr424 to Ser435) of the inner EccB_5_ (Fig. 2f). The MycP_5_–EccB_5_ interface covers a surface area of about 1,230 Å^2^, which leads to the burial of three conserved tryptophan residues (Trp437 and Trp469 of EccB_5_, and Trp523 of MycP_5_) (Fig. 2f). Additionally, loop 2 of MycP_5_ binds to the C terminus of the outer EccB_5_, which explains why a deletion of this loop previously showed reduced ESX-5 secretion in *Mycobacterium marinum*^10^ (Fig. 2d, Extended Data Fig. 6i). MycP_5_–MycP_5_ interactions are mediated mainly through loop 1 and the N-terminal extension (which run across the top of the MycP_5_ protomers), and loop 3, which contacts the neighbouring protease domain from the side (Extended Data Fig. 7c, d). Loop 5 (residues Ala151 to Val271), which is cleaved during ESX-5 maturation^11^, folds along the interface of two protease domains towards the central pore formed by the MycP_5_ trimer (Extended Data Fig. 7a). Although we could not build a complete model of loop 5 (owing to its high flexibility), this loop appears to cap the central periplasmic pore (Extended Data Fig. 7a). Notably, as loop 5 is not present in all mycosins and is dispensable for ESX-5 secretion^10^, a speculative role in gating remains to be identified. The active sites of the MycP_5_ proteases face towards the central lumen of the cavity (Fig. 2c), which implies that potential substrates of this protease are translocated through—and processed within—this periplasmic chamber.

The dimer interface between the inner and outer EccB_5_ is the largest in the periplasmic assembly, and covers a surface area of around 2,000 Å^2^ and provides a solvation-free energy gain of Δ*G* = −23 kcal mol^−1^ per dimer (Fig. 2d). By contrast, the interfaces formed between EccB_5_ dimers each bury a surface area of about 600 Å^2^, with a cumulative energy gain of only Δ*G* = −18 kcal mol^−1^ upon trimerization. This could provide an explanation as to why dimeric ESX subcomplexes are more stable than their fully assembled counterparts^6,7^. The intermolecular EccB_5_–MycP_5_ interactions (which have a surface area of around 395 Å^2^ and Δ*G* = −0.1 kcal mol^−1^) are even more modest, which provides a rationale for why interactions between MycP_5_ and the membrane complex have so far remained unknown.

## MycP_5_ stabilizes the entire membrane complex

**Extended Data Fig. 8:**
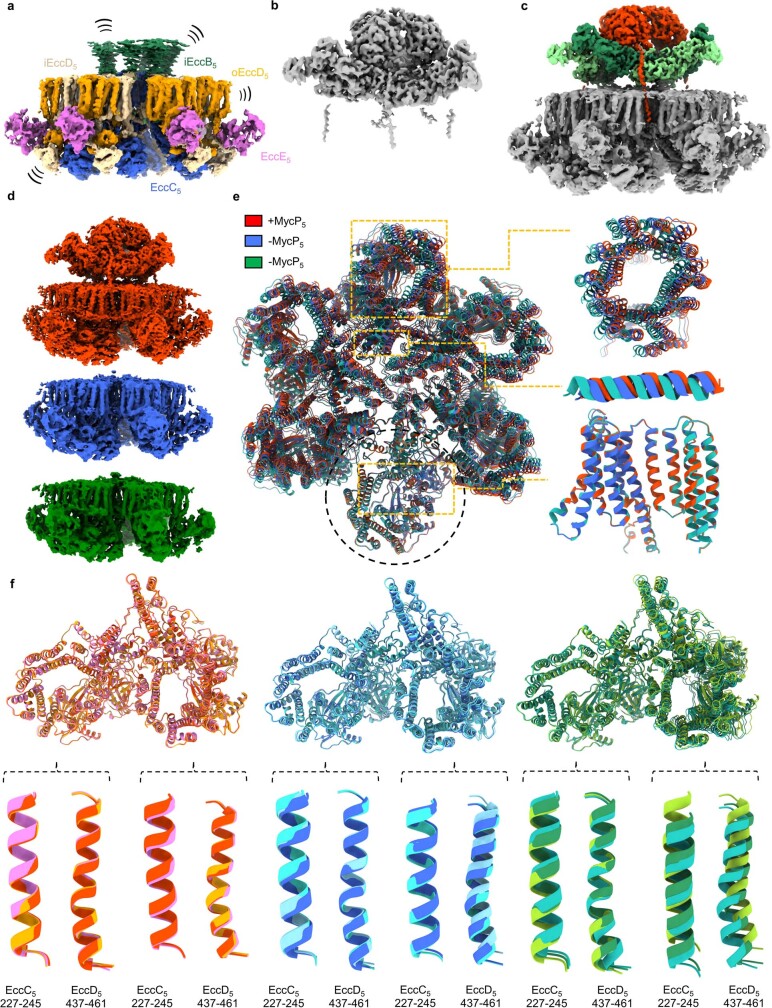
MycP_5_ drives hexamerization of periplasmic EccB_5_ and complex stability. **a**, Cryo-EM density map of a MycP_5_-free ESX-5_mtb_ membrane complex, zoned and coloured as in Fig. 1. In the absence of MycP_5_, the periplasmic domains of EccB_5_ display high flexibility. The rest of the membrane complex displays increased heterogeneity when compared to the MycP_5_-bound map. **b**, Map of difference created by subtracting the MycP_5_-free map from the MycP_5_-bound map. **c**, Overlay of **a** and **b**. **d**, MycP_5_-bound map in red and the two MycP_5_-free maps in blue and green. **e**, A model of the MycP_5_-bound map, in which MycP_5_ and residues 84–504 of EccB_5_ were removed, was fitted into the models of the two MycP_5_-free maps, as described in Methods. Models were aligned at one EccD_5_ barrel (dark dotted circle), revealing substantial variations and shifts between the three maps. Top inset shows that there is consistent variation between all three maps at the membrane level (EccD_5_ barrel). Middle inset shows variations between maps at cytosolic level (EccB_5_ N-terminal helix, residues 20–38). Bottom inset highlights inner EccD_5_ from the EccD_5_ barrel that was used for the alignment, showing that overall protomer structure does not change in the absence of MycP_5_. **f**, Dimers from every individual map, colour-coded the same as in **d** (different shades), were extracted and aligned to each other on one EccD_5_ barrel (left) as in **e**. Insets from these alignments, derived from both protomers, show that all three dimers of the MycP_5_-bound map show little to no variation, whereas the two MycP_5_-free maps show a higher degree of heterogeneity between dimers.

To further investigate the effect of MycP_5_ on the entire structure, we analysed MycP_5_-free *M. tuberculosis* ESX-5 complexes from the same preparation (Fig. 2e, g). These assemblies contained the same EccB_5_:EccC_5_:EccD_5_:EccE_5_ stoichiometry as the fully assembled complexes (Extended Data Fig. 8). Following 3D reconstruction, we obtained two MycP_5_-free *M. tuberculosis* ESX-5 maps that displayed resolution estimates of about 4.5 and about 6.7 Å (Extended Data Fig. 4). The differences were most notable on the periplasmic side, on which the six EccB_5_ copies showed high flexibility and did not form a stable triangular scaffold in the absence of MycP_5_ (Fig. 2e). This shows that MycP_5_ enables the trimerization of the EccB_5_ dimers in the periplasm. This result is highly interesting, because mycosins are subtilisin-like proteases without any additional domains apart from a TMH, some small loops and an N-terminal extension that wraps around the protein^12,13^. A more structural role for mycosin has previously been predicted^8^ but we now understand the essential role of mycosins in T7SSs.

**Extended Data Fig. 9:**
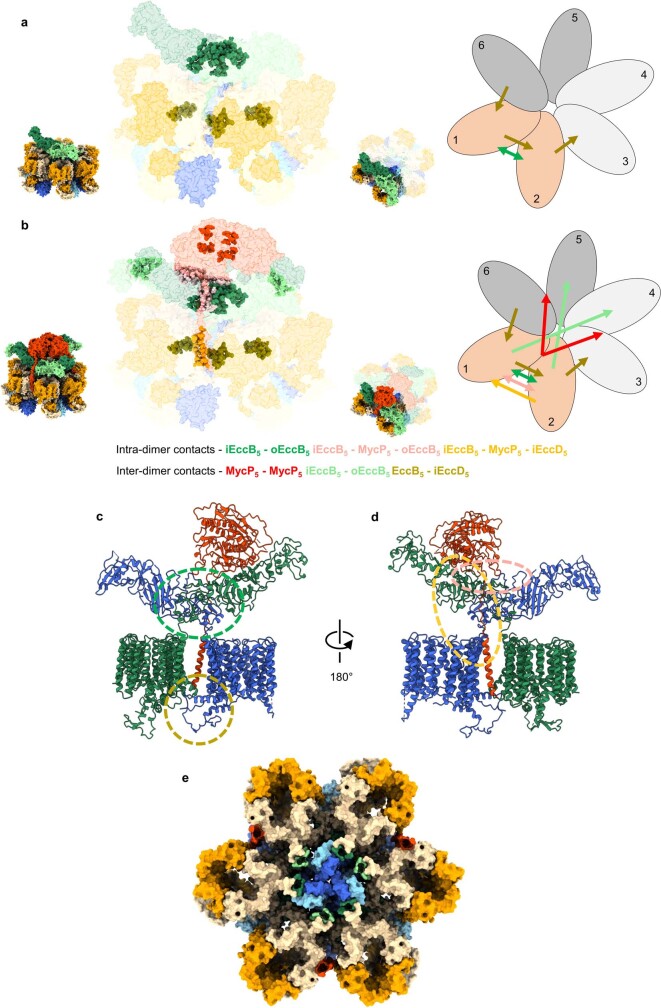
MycP_5_ creates more interaction points between protomers and dimers. **a**, Transparent surface model of a MycP_5_-free map with one EccB_5_ dimer at the periplasmic side, highlighting the interfaces of the protomers from a dimer. In the absence of MycP_5_, the two protomers from within one dimer exhibit two interactions: an EccB_5_–EccB_5_ interaction between their periplasmic domains, and a cytosolic one between EccB_5_–inner EccD_5_. The dimer further contacts the two immediate protomers of adjacent dimers through the mentioned EccB_5_–EccD_5_ cytosolic interaction. **b**, Transparent surface model of the MycP_5_-bound map, highlighting the interface of protomers from a dimer. On top of the mentioned contacts, in the presence of MycP_5_, protomers from a dimer interact with each other at the periplasmic side through inner EccB_5_–MycP_5_–outer EccB_5_, while MycP_5_ further anchors the periplasmic assembly to the stable EccD_5_ raft through EccB_5_–MycP_5_–inner EccD_5_. MycP_5_ also guides dimer–dimer interactions. By stabilizing the three EccB_5_ dimers in the triangle assembly, MycP_5_ promotes inner EccB_5_–outer EccB_5_ interactions between opposing protomers from adjacent dimers. Additionally, MycP_5_ promotes dimer–dimer contacts through MycP_5_–MycP_5_ interactions in the periplasm. Colour-coded legend applies to **a**, **b**. **c**, **d**, Inside (**c**) and outside (**d**) view of a dimer containing EccB_5_, MycP_5_ and the TMHs of EccD_5_. For purposes of clarity, one EccD_5_–EccB_5_ protomer is coloured in blue, and the second protomer is in green and the MycP_5_ in red. Interactions between protomers of a dimer are highlighted and colour-coded as in **a**, **b**. **e**, Top view of a surface model missing the periplasmic domains of EccB_5_ and MycP_5_. The planes of protomers in which MycP_5_ binds inner EccD_5_ are tilted by about 5° compared to the MycP_5_-unbound ones.

In contrast to the periplasmic domain, the cytosolic and membrane regions of the MycP_5_-free maps were more similar to those of the MycP_5_-containing particles (Extended Data Fig. 8). However, the MycP_5_-free particles displayed an increased heterogeneity that affected the entire complex, which resulted in a slight waving of the membrane region and an increased angle variation between individual protomers (Fig. 2g, Extended Data Fig. 8, Supplementary Video 4). The protease domain and TMH of MycP_5_ synergistically reinforce the membrane complex. Their interactions with periplasmic inner EccB_5_ and membrane-embedded outer EccD_5_ (from separate protomers within a dimer) better anchor the periplasmic assembly to the membrane, while also stabilizing the dimeric unit (Extended Data Figs. 8f, 9). Additionally, by driving the formation of the periplasmic assembly, MycP_5_ stabilizes the entire complex by promoting cross-dimer MycP_5_–MycP_5_ and inner EccB_5_–outer EccB_5_ interactions (Extended Data Fig. 9). Our MycP_5_-free *M. tuberculosis* ESX-5 maps are reminiscent of the hexameric, low-resolution negative-stain structure of ESX-5 from *Mycobacterium xenopi*, in which the periplasm was similarly disorganized in the absence of MycP_5_^5^.

## EccC_5_ gates a potential secretion conduit

**Extended Data Fig. 10:**
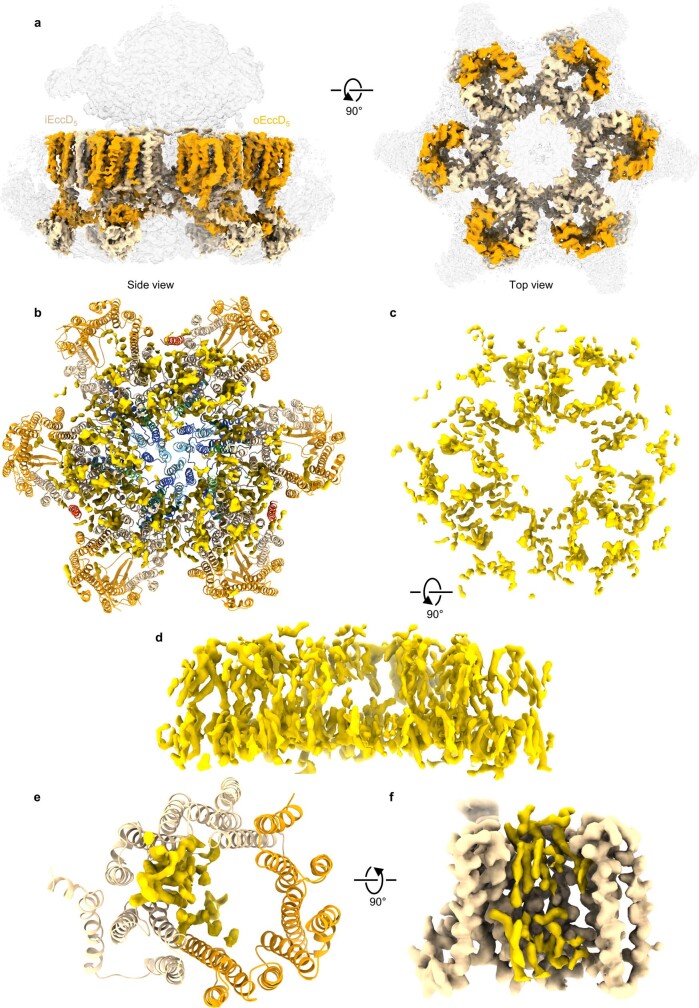
Six lipid-filled EccD_5_ barrels form a central raft. **a**, Side and top view of an intact ESX-5_mtb_ assembly, in which inner EccD_5_ and outer EccD_5_ are coloured as in Fig. 1 and the rest of the components are transparent. **b**, Top view of the membrane region of the ESX-5_mtb_ model overlaid with observed lipids, coloured in bright yellow. **c**, Same view as **b**, but showing only the lipids. **d**, Same image as **c**, but rotated 90° to show a side view, highlighting a bilayer-like structure. **e**, Top view of an EccD_5_ barrel with observed lipids bound to inner EccD_5_. **f**, Side view of an inner EccD_5_ monomer displayed as zoned density and overlaid with observed lipids.

At the membrane level, six EccD_5_-dimer barrels (each of which contains 22 TMHs) together form a circular raft with an inner cavity (Extended Data Fig. 10). Within this raft, inner EccD_5_ monomers are situated closer to the centre, whereas outer EccD_5_ monomers face towards the periphery of the membrane complex. The EccD_5_ membrane barrels are structurally highly similar to the homologous EccD_3_ barrel in the ESX-3 subassembly^6,7^. The inner surface of each EccD_5_ barrel is decorated with densities that are attributable to stably bound lipids or detergent molecules, which suggests that, in their native membrane environment, these barrels are filled with membrane lipids (Extended Data Fig. 10e, f).

**Fig. 3:**
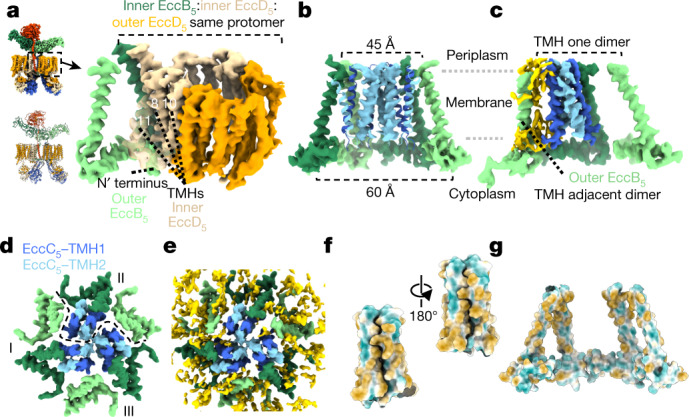
A basket formed by the EccB_5_ TMHs holds three four-TMH bundles of EccC_5_. **a**, Angled view from the outside of the complex, showing the TMH and N terminus of an outer EccB_5_ interacting with a pocket formed by TMH8, TMH10 and TMH11 of inner EccD_5_ from the adjacent barrel. **b**, Side cross-section through the EccB_5_ basket that contains the EccC_5_ TMH bundles. Light blue densities depict the three copies of EccC_5_ TMH2 that form the central pyramid. Two TMHs of EccC_5_ were removed for clarity. Sizes indicate the inner diameters of the EccB_5_ basket. **c**, Side cross-section through an EccB_5_ basket, showing that the EccC_5_ TMH bundle does not interact with outer EccB_5_ from its own dimer, but instead forms lipid-mediated interactions with the outer EccB_5_ TMH of the adjacent dimer. Lipids are shown in gold. **d**, Top view of the central EccB_5_ basket and the EccC_5_ TMH bundles. Dashed line marks the TMHs that belong to one dimeric unit. **e**, As in **d**, highlighting the lipid-rich environment. In the central area that surrounds the EccC_5_ pyramid, lipids are not clearly distinguishable (which suggests fluidity in this area). **f**, **g**, Surface model displaying the hydrophobicity of an EccC_5_ TMH bundle (**f**) and the EccB_5_ basket (**g**). Hydrophilic amino acids are shown in turquoise, and hydrophobic residues are shown in sepia.

The TMH of each copy of EccB_5_ is anchored within the confinement of the EccD_5_ raft through hydrophobic interfaces that are provided by TMH6 and TMH11 of inner EccD_5_ and stably bound lipids (Fig. 3a, Extended Data Fig. 6f). Notably, no substantial intermolecular interactions can be found between adjacent EccD_5_ barrels. Instead, coupling between two neighbouring EccD_5_ barrels is achieved by the N-terminal loop and α-helix of EccB_5_ that run parallel to the cytoplasmic side of the inner membrane and engage in interactions with the TMHs of the neighbouring EccD_5_ barrel in a clockwise manner (Fig. 3a, Extended Data Fig. 6g, h). Because the TMH of EccB_5_ is slightly angled towards the centre of the complex, the architecture of the hexamer of EccB_5_ TMHs is reminiscent of a basket, the inner diameter of which shrinks from around 60 Å to around 45 Å towards the periplasmic side (Fig. 3b).

**Extended Data Fig. 11:**
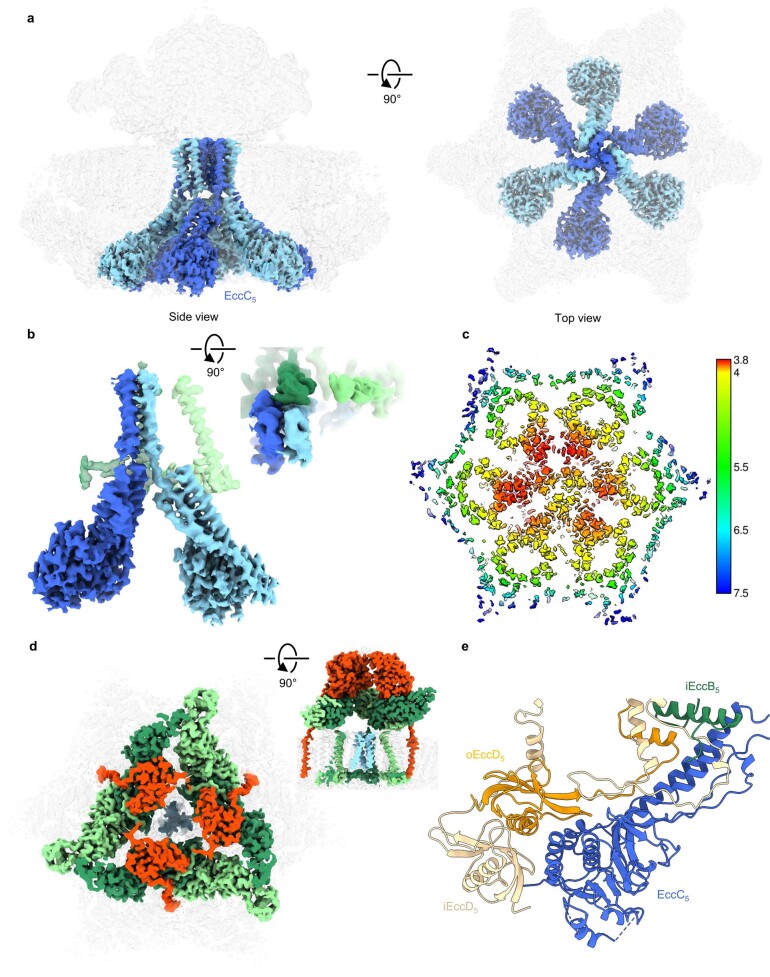
Three four-TMH-bundles of EccC_5_ gate a central pore. **a**, Side and top view of an intact ESX-5_mtb_ assembly, in which EccC_5_ is coloured in alternating light and dark blue and the rest of the components are transparent. **b**, Extracted dimeric EccC_5_ and the TMHs and N termini of EccB_5_ from the same dimer. The 90° inset rotation shows that the TMH of outer EccB_5_ is not contacted by the TMHs of EccC_5_. **c**, Top membrane cross-section through a local-resolution map of a C1 full-complex reconstruction, displaying decreased resolution of the central space occupied by the TMHs of EccC_5_, compared to the surrounding EccB_5_ basket and TMHs of inner EccD_5_. **d**, Top view of the full membrane complex with the EccC_5_ TMH pyramid in light blue and the periplasmic EccB_5_–MycP_5_ in the same colours as in Fig. 1. The EccC_5_ TMH pyramid aligns with the periplasmic cavity and the MycP_5_-formed pore. MycP_5_ top part is partially sectioned, for clarity. Inset showing a 90° rotation side cross-section of the same map. **e**, Ribbon model highlighting the structural features of the cytosolic bridge.

EccC is the only component that is present in all T7SSs (including in related systems in Firmicutes^2^), and is therefore thought to be the central component in this nanomachinery. Each EccC protein has—in addition to two TMHs—four FtsK/SpoIIIE-like ATPase domains (also known as nucleotide-binding domains (NBDs)) that are known to be important for secretion^6,7,14^. We fully resolved the twelve EccC_5_ TMHs in the intact *M. tuberculosis* ESX-5 complex; these form three four-TMH bundles, each of which belongs to the EccC_5_ molecules of one dimer (Fig. 3b–d, Extended Data Fig. 11). These bundles are held together by hydrophobic interactions and effectively seal the central space of the membrane complex, which is enclosed by the EccB_5_ basket (Fig. 3d, Supplementary Video 3). Two EccC_5_ TMHs from each bundle contact the TMH of the inner EccB_5_, which leaves the outer EccB_5_ TMH unbound by EccC_5_ (Fig. 3c, d). At the very centre of the complex, one TMH of each bundle contributes to the formation of a pyramidal assembly that aligns with the periplasmic chamber (Extended Data Fig. 11d).

The chamber within the EccB_5_ basket appears to be filled with lipids. However, the density for these lipids is more ambiguous than that of the lipids in and around the EccD_5_ barrels, which suggests that the lipids within this chamber are more fluid (Fig. 3e). Notably, the local resolution gradually increases when moving from the centre to the EccB_5_ basket, where the resolution is highest (Extended Data Fig. 11c). This indicates that the EccC_5_ TMH bundles display more flexibility, as compared to the rigid EccB_5_ basket. The entrance to the putative EccC_5_ pore widens on the cytoplasmic side, where the EccC_5_ stalk domains expand radially (Extended Data Figs. 5, 11a). Together, our data suggest that the six EccD_5_ barrels provide a stable scaffold for assembly of a secretion pore that is confined by the EccB_5_ TMHs and gated through three EccC_5_ TMH bundles. Secretion through the inner membrane complex would require rearrangement of the EccC_5_ TMHs. Such a proposed central pore would extend into the periplasmic chamber that is formed by EccB_5_ and MycP_5_.

## Cytosolic EccC_5_ adopts two conformations

At the cytoplasmic side of the complex, EccC_5_ has a stalk helix that connects its second TMH to the first NBD (which is also known as the DUF domain). This NBD is bound to the cytosolic domains of inner and outer EccD_5_, which—in turn—are bound to EccE_5_ at the periphery, together forming a ‘cytosolic bridge’ (Fig. 1f).

**Fig. 4:**
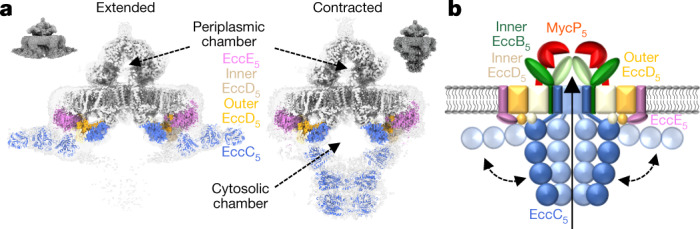
EccC_5_ adopts an extended and a contracted conformation. **a**, Side cross-section of density maps, showing the extended and contracted conformation of EccC_5_. The periplasmic and cytoplasmic chambers formed by EccB_5_–MycP_5_ and by EccC_5_ upon closing are highlighted. Homology models of the three C-terminal NBDs of EccC_5_ are fitted in the cytosolic densities. Cytosolic bridge components are coloured as in Fig. 1. **b**, Model of the intact T7SS inner-membrane complex, highlighting the two conformations of EccC_5_.

**Extended Data Fig. 12:**
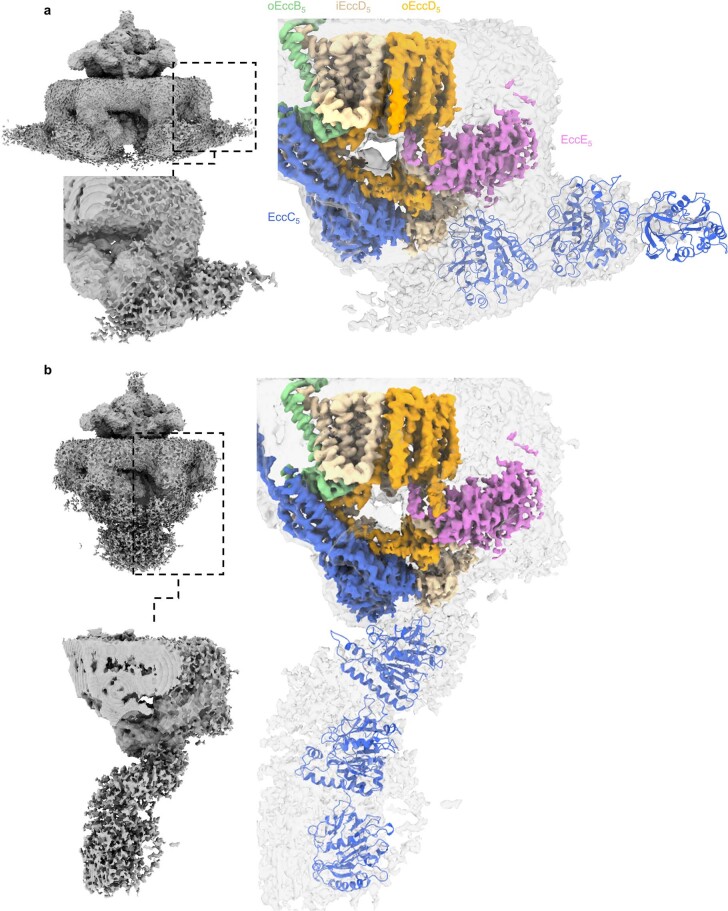
EccC_5_ adopts two separate conformations. **a**, Extended conformation in which an EccC_5_ NBD1–NBD2–NBD3 model is fitted to highlight the overall position of these domains with respect to the rest of the membrane complex. **b**, As in **a**, but for the contracted conformation.

The distal C-terminal part of EccC_5_, which comprises a string of three NBDs (NBD1, NBD2 and NBD3), adopts two main conformations: we refer to these as extended and contracted (Fig. 4a). In the extended state, the C-terminal three NBDs of EccC_5_ bend parallel to the membrane, and align with the cytosolic domains of inner EccD_5_ and EccE_5_ of the same protomer, and extend beyond the diameter of the membrane assembly. Although of considerably lower resolution, this density can confidently accommodate a homology model that consists of the three EccC_5_ NBD domains (Fig. 4a, Extended Data Fig. 12). Further classification of the extended state reveals EccC_5_ to be more heterogenous beyond NBD1, which suggests that NBD1 is more stably bound to components of its own protomer. Although we found only a relatively small number of particles in the contracted conformation, we solved the stable core of the membrane complex to sub-nanometre resolution (Extended Data Fig. 3). In the contracted state, the flexible arms of EccC_5_ extend from the interface between the DUF domain of EccC_5_ and the cytosolic domain of inner EccD_5_ (Fig. 4a, Extended Data Fig. 12). We observed three separate disc-like structures that gradually constrict from the top to the bottom. This density shows a gap at the interface between NBD1 and NBD2. This would allow the previously postulated^14,15^ binding of substrates to the linker 2 that connects NBD1 and NBD2, resulting in the displacement of this linker and the activation of NBD1. The highly dynamic cytoplasmic domains of the machinery may provide the basis for substrate selection, recognition or transport across the membrane.

Our work provides a fully assembled structure of the ESX-5 inner-membrane complex of *M. tuberculosis*. As the membrane components of the five mycobacterial ESX systems show high sequence conservation, our results probably constitute a general structural blueprint for all of these T7SSs—including the virulence-related ESX-1 system (Supplementary Figs. 2, 3). Furthermore, our structure will serve as a platform for the identification of interactions that—if perturbed by small molecules—would aid in the treatment of tuberculosis.

## Methods

No statistical methods were used to predetermine sample size. The experiments were not randomized, and investigators were not blinded to allocation during experiments and outcome assessment.

### Molecular biology

*Escherichia coli* Dh5α was grown at 37 °C and 200 rpm in LB medium supplemented with 30 μg ml^−1^ streptomycin. Cloning was performed in *E. coli* Dh5α using IProof DNA polymerase from BioRad and restriction enzymes from New England Biolabs. A list of the primers used for amplification is available in Supplementary Table 3.

The plasmid expressing *M. tuberculosis* ESX-5 was built as follows: the backbone of the previously described pMV ESX-5_mxen_ plasmid^5^ was modified to encode the unique restriction sites DraI and PacI upstream and SpeI and NdeI downstream of the TwinStrep tag sequence. The *rv1782–rv1783* (*eccB*_*5*_*–eccC*_*5*_) region, including about 380 bp upstream of *eccB*_*5*_, of *M. tuberculosis* H37Rv was amplified while adding DraI and PacI restriction sites at the 5′ and 3′ ends, respectively (primers 1 and 2), and cloned into the modified plasmid upstream of the TwinStrep tag sequence, resulting in plasmid intermediate 1. The *M. tuberculosis* H37Rv region spanning *rv1791–rv1798* (*pe19–eccA*_*5*_) was amplified while adding SpeI and NdeI unique restriction sites (primers 3 and 4) and cloned downstream of the TwinStrep tag sequence into the intermediate 1, resulting in plasmid intermediate 2. Plasmid intermediate 2 was digested with SpeI and SnaBI, removing the region *rv1791–rv1794* (*pe19–espG*_*5*_*)*, and the region encompassing *rv1785–rv1794* (*cyp143–espG*_*5*_) was amplified as two individual PCR products (primers 5 and 6 and primers 7 and 8). The restricted backbone and PCR products were InFusion (Takara Bio)-ligated, resulting in the final pMV-ESX-5_mtb_ containing the entire *rv1782–rv1798* (*eccB*_*5*_*–eccA*_*5*_) locus.

### Isolation of mycobacterial cell envelopes

*Mycobacterium smegmatis* MC^2^155 expressing *M. tuberculosis* ESX-5 was grown at 37 °C and 90 rpm in LB medium supplemented with 0.05% Tween 80 and 30 μg ml^−1^ streptomycin. Cultures were grown to an optical density (OD) at 600 nm of about 1.5, spun down for 15 min at 12,000*g* in a JLA-8.1000 rotor and subsequently washed in PBS. After culture collecting, all subsequent steps were performed at 4 °C. Washed cell pellets were resuspended in buffer A (50 mM Tris-HCl pH 8, 300 mM NaCl and 10% glycerol) at a concentration of about 50 OD ml^−1^ and lysed by passing two times through a high-pressure homogenizer (Stansted) using a pressure of 0.83 kbar. Unbroken cells were pelleted at 5,000*g* for 5 min and supernatants were transferred to ultracentrifugation tubes. Cell envelopes were separated from the soluble fraction by ultracentrifugation at 150,000*g* for 1.5 h. Following ultracentrifugation, supernatants were discarded, pellets were washed once with buffer A, resuspended in buffer A at a concentration of 750–1,000 OD ml^−1^, snap-frozen in liquid nitrogen and stored at −80 °C until further use. The protein concentration of the cell envelope fraction was measured by BCA assay (Pierce).

### Purification of the *M. tuberculosis* ESX-5 membrane complex

All steps were performed at 4 °C. The *M. tuberculosis* ESX-5 was purified as follows: cell envelope fractions were diluted to 3 mg ml^−1^ in buffer B (50 mM Tris-HCl pH 8, 300 mM NaCl and 5% glycerol), supplemented with 0.25% DDM, 3 mM ADP–AlF_3_ and 6 mM MgCl_2_. Following solubilization, the cell envelope mixture was spun down at 100,000*g* for 20 min, supernatants were collected and incubated with StrepTactin resin (IBA). Beads were subsequently washed with buffer B supplemented with 0.03% DDM, 1 mM ADP–AlF_3_ and 2 mM MgCl_2_. Bound protein was eluted from the resin with buffer B supplemented with 0.03% DDM, 3 mM ADP–AlF_3_, 6 mM MgCl_2_ and 10 mM desthiobiotin. The protein concentration of the eluate was measured by Bradford assay and amphipol A8-35 was added in an amphipol:protein ratio of 5:1. After a 1-h incubation, the amphipol-containing eluate was incubated overnight (around 12–16 h) with BioBeads in a BioBeads:detergent ratio of 20:1. Subsequently, BioBeads were removed using gravity flow chromatography columns and the sample was concentrated using Amicon Ultra 0.5-ml 100-kDa spin concentrators. The concentrated sample was further purified through size exclusion chromatography (SEC), using a Superose 6 Increase column running in buffer C (20 mM Tris-HCl pH 8, 200 mM NaCl) supplemented with 1 mM ADP–AlF_3_ and 2 mM MgCl_2_. Size exclusion chromatography fractions were analysed by blue-native polyacrylamide gel electrophoresis (BN-PAGE) and negative-stain electron microscopy, after which the appropriate fractions were concentrated for cryo-EM using Amicon Ultra 0.5-ml 100-kDa spin concentrators. The initial Arctica sample was purified similarly, with the addition of 5% glycerol in the SEC purification step and the omission of ADP–AlF_3_ and MgCl_2_ from the purification protocol.

### BN-PAGE

Samples consisting of either solubilized membranes or purified membrane complexes were mixed with 5% G-250 sample additive (Invitrogen), to a final concentration of about 0.2%, and run on 3–12% NativePage Bis-Tris Protein Gels (Invitrogen) according to manufacturer specifications. Gels were either stained with Coomasie R-250 or transferred to PVDF membranes and stained with appropriate antibodies (Supplementary Fig. 1). Antisera against EccB_5_, used in Extended Data Fig. 1b, was raised against the synthetic peptide CLPMDMSPAELVVPK and has previously been described^16^. Polyclonal rabbit antisera against the peptide was raised in rabbits by Innovagen using Stimune (Prionix) as adjuvants. The antibody was used as a 1:5,000 dilution. Blots were visualized on a ChemoStar TouchMotionCor2 using ChemoStarTS.

### Negative-stain electron microscopy

Carbon-coated copper grids were glow-discharged for 30 s at 25 mA using a GloQube Plus Glow Discharge System (Electron Microscopy Sciences). Four microlitres of diluted sample was applied to the grids and incubated for 30 s. The sample was blotted off from the side and the grid was washed briefly with 4 μl of staining solution (2% uranyl acetate) and then stained with 4 μl of the staining solution for 30 s. The stain was blotted off from the side and grids were air-dried. Grids were imaged using a Thermo Fisher Scientific Talos L120C TEM equipped with a 4K Ceta CEMOS camera using TIA 4.1.5.

### Cryo-EM sample preparation

For the main datasets, purified sample was applied to Quantifoil R2/2, 200 mesh, copper grids floated with an additional approximately 1.1-nm layer of amorphous carbon. Four microlitres of sample was applied onto glow-discharged grids (30 s at 25 mA) and allowed to disperse for 60 s at 4 °C and 100% humidity. Grids were blotted for 4–6 s with a blot force of −5 and plunge-frozen in a liquid propane–ethane mixture, using a Thermo Fisher Scientific Vitrobot Mark V. For the initial Arctica dataset, all steps were similar, with the exception of the additional layer of amorphous carbon.

### Cryo-EM data acquisition

The initial cryo-EM dataset was collected on a 200-kV FEI Talos Arctica electron microscope equipped with a Falcon III direct electron detector running in counting mode and using Thermo Fisher Scientific EPU 1.11. A total of 853 movies were recorded with a nominal magnification of 150,000×, corresponding to a pixel size of 0.96 at the specimen level. Movies were recorded with a total dose of 40.28 electrons per A^2^, fractionated in 38 frames over a 40-s exposure time and with a nominal defocus range of 1–2.5 μm.

The two high-resolution datasets were recorded using Thermo Fisher Scientific EPU 2.4 software on a 300-kV Titan Krios TEM, equipped with a Gatan K3 direct electron detector running in counting mode and a Gatan Bioquantum energy filter (slit size 10 eV). We recorded 7,984 and 9,389 movies in counting mode in the two separate sessions with a nominal magnification of 81,000×, corresponding to a pixel size of 1.1 Å at the specimen level. Movies were recorded with a total dose of 59.5 electrons per A^2^, fractionated in 50 frames over a 3-s exposure time and with a nominal defocus range of 1–3 μm.

### Cryo-EM data processing

Single-particle analysis was performed using Relion3.1^17^, unless stated otherwise. For the initial Arctica dataset, movies were motion-corrected using MotionCor2^18^ and dose-weighted, and the contrast-transfer function (CTF) was estimated using CTFFIND4^19^. Automated particle-picking was performed using Cryolo^20^ and the pretrained Janni model. Following particle extraction and several rounds of 2D classification to remove obvious artefacts, an initial de novo model was generated. The dataset was further cleaned using 3D classification and the best class was subsequently used for reference-based particle-picking. Following 2D and 3D classification (and 3D refinement in C1), the map displayed an apparent threefold symmetry and was further refined in C3. This final map displayed an estimated 13.5 Å resolution.

In the first Krios dataset, movies were motion-corrected using MotionCor2, dose-weighted and the CTF was estimated using CTFFIND4. Automated particle-picking was performed using Cryolo with the pretrained Janni model and a low threshold. Particles were extracted and binned 4× and several rounds of 2D classification were performed followed by 3D classification with the 30 Å-filtered Arctica model as a template. The resulting particles were re-extracted without binning, CTF-corrected and polished and refined in C1, giving a map with an estimated overall resolution of 4.5 Å. For the cytosolic region, particles were recentred on the cytosolic region, re-extracted, CTF-corrected, polished and 3D-refined. Following refinement, the density accounting for individual cytosolic dimers was subtracted, resulting in a particle stack that was three times larger. Cytosolic dimers were first refined with a mask encompassing both cytosolic bridges. Subsequently, these were focus-refined using a soft mask around one of the cytosolic bridges. This map was refined using the default Relion value ‘--tau2fudge 2’ but also ‘--tau2fudge 4’, which increased the overall connectivity of the lower cytosolic area. The final map for the cytosolic bridge showed an estimated resolution of 3.3 Å and was sharpened using either Relion postprocessing or DeepEMhancer^21^. DeepEMhancer further helped to improve the observed anisotropy, overall map connectivity.

The second Krios dataset was processed similarly, with some exceptions. Following 3D classification of the binned data against the 4.5 Å Krios map filtered to 30 Å, the two maps with and without MycP_5_ were processed separately. The MycP_5_-unbound map displayed increased heterogeneity and—following unbinned re-extraction and refinement—the particles were 3D-classified without alignment, resulting in two obvious classes of 4.5 Å and 6.7 Å resolution. Model free density modification in Phenix.Resolve_Cryo_EM^22^ further improved the resolution to 4.3 Å and 5.8 Å, respectively. By contrast, a similar 3D classification for the MycP_5_-bound map did not further classify into structurally distinct classes. Following unbinned re-extraction and refinement, the MycP_5_-bound map showed an overall resolution of 4 Å, which was further improved to 3.8 Å after C3 refinement. Model free density modification in Phenix.Resolve_Cryo_EM further improved the resolution of the entire C1 map to 3.8 Å and of the C3 refined map to 3.56 Å. For the periplasmic map, the centre of mass for that region was determined using Chimera^23^ and the particles were recentred, extracted, 3D-refined and polished to obtain the periplasmic map at an estimated 3.8 Å resolution in C1. Following 3D classification without alignment and further refinement in C3, the estimated resolution of the periplasmic map improved to 3.5 Å. To separate the two states of EccC_5_, particles were recentred on the lower cytosolic region, at the level of the DUF domain, polished and 3D-refined. This was followed by a masked 3D classification in which the mask contained NBD1 and NBD2 of EccC_5_ in the extended conformation. The two main classes were further 3D-refined unmasked and subsequently masked, leading to a map of about 4.27 Å for the extended conformation and 7.6 Å for the contracted conformation.

### Model building and refinement

Model building started by generating homology models for MycP_5_, EccB_5_, EccC_5_ and EccD_5_ with Phyre2^24^. For MycP_5_, Protein Data Bank (PDB) entry 4J94^12^ served as a structural template, and PDB entries 4KK7^25^, 4NH0^14^ and 6SGW^6^, and 6SGZ^6^ served as reference models for EccB_5_, EccC_5_ and EccD_5_, respectively. To obtain atomic models of the periplasmic part (MycP_5_–EccB_5_) of the *M. tuberculosis* ESX-5 complex, homology models of MycP_5_ and EccB_5_ were rigid-body-fitted into a C1 symmetry, focus-refined periplasmic *M. tuberculosis* ESX-5 map (Electron Microscopy Data Bank (EMDB) code EMD-12518) using the fit-in-map tool in ChimeraX (v.1.0)^26^. Model building, extension and interactive refinement was performed with ISOLDE (v.1.0.1)^27^, a molecular-dynamics-guided structure refinement tool within ChimeraX (v.1.0). The resulting coordinate file (PDB 7NPS) was further refined with Phenix.real_space_refine (v.1.18.2-3874)^28^ using reference model restraints, strict rotamer matching and disabled grid search. Model validation was carried out using the MolProbity web server^29^ and EMRinger^30^ within the Phenix software package. Models for the membrane-embedded region (MycP_5_–EccB_5_–EccC_5_–EccD_5_) and cytoplasmic bridge (cytosolic domains of EccC_5_–EccD_5_) (PDB 7NPT) were built in the same way, using a reconstruction of the full *M. tuberculosis* ESX-5 complex (EMDB EMD-12517) and a focus-refined map of the cytoplasmic domains (EMDB EMD-12520) sharpened with DeepEMhancer, respectively. Finally, a composite model was assembled by fusing the periplasmic assembly and six copies of the cytosolic bridge to the membrane-embedded region model. This composite model was then refined against the full *M. tuberculosis* ESX-5 complex map with C1 symmetry (PDB 7NP7 and EMDB EMD-12514) and C3 symmetry (PDB 7NPR and EMDB EMD-12517).

Modelling into MycP_5_-free maps was performed with ISOLDE using the composite ESX-5 model, in which MycP_5_ and the periplasmic domain of EccB_5_ (residues 84–507) had been deleted. Adaptive distance restraints as well as torsion restraints were applied to all atoms to restrain short-range conformational changes but allow for long-range conformational movements. ISOLDE simulations for dynamic fitting of the coordinate file into EMD-12521 and EMD-12522 were performed (about 10 min, 10 K) after which the models showed satisfying fits to the new maps without further manual intervention. MycP_5_-free models were further refined against the maps using Phenix.real_space_refine (v.1.18.2-3874) as stated.

Visualization of atomic coordinates and map volumes was performed with ChimeraX (v.1.1) and PyMOL v.2.40^31^. Buried surface areas between subunits were calculated by PISA^32^.

### Reporting Summary

Further information on research design is available in the Nature Research Reporting Summary linked to this article.

## Online content

Any methods, additional references, Nature Research reporting summaries, source data, extended data, supplementary information, acknowledgements, peer review information; details of author contributions and competing interests; and statements of data and code availability are available at 10.1038/s41586-021-03517-z.

## Supplementary information

Supplementary Information This file contains Supplementary Tables 1-3 and Supplementary Figures 1-3.

Reporting Summary.

Peer Review File

Video 1 Overall structure of the ESX-5mtb membrane complex.

Video 2 The periplasmic dome of the ESX-5mtb membrane complex.

Video 3 The central, EccC5 gated, pore of the ESX-5mtb membrane complex.

Video 4 Flexibility of the MycP5 free maps and stabilization upon MycP5 binding.

## Data Availability

Cryo-EM maps have been deposited in the EMDB under accession codes EMD-12514 (full complex in C1), EMD-12517 (full complex in C3), EMD-12518 (periplasmic map in C1), EMD-12519 (periplasmic map in C3), EMD-12520 (cytosolic bridge), EMD-12521 (MycP_5_-free map 1), EMD-12522 (MycP_5_-free map 2), EMD-12523 (EccC_5_, extended state) and EMD-12525 (EccC_5_, contracted state). The composite model settled in the C1 and C3 full maps, periplasm in C1, cytosolic bridge, MycP_5_-free map 1 and MycP_5_-free map 2 have been deposited in the PDB under accession codes 7NP7, 7NPR, 7NPS, 7NPT, 7NPU and 7NPV, respectively. All other data are available from the corresponding author upon reasonable request.

## References

[1] WHO. *Global Tuberculosis Report* (WHO, 2020).

[2] Bunduc CM, Bitter W, Houben ENG (2020). Structure and function of the mycobacterial type VII secretion systems. Annu. Rev. Microbiol..

[3] Gröschel MI, Sayes F, Simeone R, Majlessi L, Brosch R (2016). ESX secretion systems: mycobacterial evolution to counter host immunity. Nat. Rev. Microbiol..

[4] Rybniker J (2014). Anticytolytic screen identifies inhibitors of mycobacterial virulence protein secretion. Cell Host Microbe.

[5] Beckham KS (2017). Structure of the mycobacterial ESX-5 type VII secretion system membrane complex by single-particle analysis. Nat. Microbiol..

[6] Famelis N (2019). Architecture of the mycobacterial type VII secretion system. Nature.

[7] Poweleit N (2019). The structure of the endogenous ESX-3 secretion system. eLife.

[8] van Winden VJC (2016). Mycosins are required for the stabilization of the ESX-1 and ESX-5 type VII secretion membrane complexes. MBio.

[9] Xie XQ (2016). Crystallographic observation of the movement of the membrane-distal domain of the T7SS core component EccB_1_ from *Mycobacterium tuberculosis*. Acta Crystallogr. F.

[10] van Winden VJC, Damen MPM, Ummels R, Bitter W, Houben ENG (2019). Protease domain and transmembrane domain of the type VII secretion mycosin protease determine system-specific functioning in mycobacteria. J. Biol. Chem..

[11] van Winden VJC, Bunduc CM, Ummels R, Bitter W, Houben ENG (2020). A chimeric EccB–MycP fusion protein is functional and a stable component of the ESX-5 type VII secretion system membrane complex. J. Mol. Biol..

[12] Solomonson M (2013). Structure of the mycosin-1 protease from the mycobacterial ESX-1 protein type VII secretion system. J. Biol. Chem..

[13] Wagner JM (2013). Understanding specificity of the mycosin proteases in ESX/type VII secretion by structural and functional analysis. J. Struct. Biol..

[14] Rosenberg OS (2015). Substrates control multimerization and activation of the multi-domain ATPase motor of type VII secretion. Cell.

[15] Bunduc CM, Ummels R, Bitter W, Houben ENG (2020). Species-specific secretion of ESX-5 type VII substrates is determined by the linker 2 of EccC_5_. Mol. Microbiol..

[16] Houben EN (2012). Composition of the type VII secretion system membrane complex. Mol. Microbiol..

[17] Zivanov J, Nakane T, Scheres SHW (2020). Estimation of high-order aberrations and anisotropic magnification from cryo-EM data sets in *RELION*-3.1. IUCrJ.

[18] Zheng SQ (2017). MotionCor2: anisotropic correction of beam-induced motion for improved cryo-electron microscopy. Nat. Methods.

[19] Rohou A, Grigorieff N (2015). CTFFIND4: fast and accurate defocus estimation from electron micrographs. J. Struct. Biol..

[20] Wagner T, Raunser S (2020). The evolution of SPHIRE-crYOLO particle picking and its application in automated cryo-EM processing workflows. Commun. Biol..

[21] Sanchez-Garcia, R. et al. DeepEMhancer: a deep learning solution for cryo-EM volume post-processing. Preprint at 10.1101/2020.06.12.148296 (2020). 10.1038/s42003-021-02399-1 PMC8282847 34267316

[22] Terwilliger TC, Ludtke SJ, Read RJ, Adams PD, Afonine PV (2020). Improvement of cryo-EM maps by density modification. Nat. Methods.

[23] Pettersen EF (2004). UCSF Chimera—a visualization system for exploratory research and analysis. J. Comput. Chem..

[24] Kelley LA, Mezulis S, Yates CM, Wass MN, Sternberg MJ (2015). The Phyre2 web portal for protein modeling, prediction and analysis. Nat. Protocols.

[25] Wagner JM (2016). Structures of EccB1 and EccD1 from the core complex of the mycobacterial ESX-1 type VII secretion system. BMC Struct. Biol..

[26] Pettersen EF (2021). UCSF ChimeraX: structure visualization for researchers, educators, and developers. Protein Sci..

[27] Croll TI (2018). ISOLDE: a physically realistic environment for model building into low-resolution electron-density maps. Acta Crystallogr. D.

[28] Liebschner D (2019). Macromolecular structure determination using X-rays, neutrons and electrons: recent developments in Phenix. Acta Crystallogr. D.

[29] Williams CJ (2018). MolProbity: more and better reference data for improved all-atom structure validation. Protein Sci..

[30] Barad BA (2015). EMRinger: side chain-directed model and map validation for 3D cryo-electron microscopy. Nat. Methods.

[31] The PyMOL Molecular Graphics System v.1.8 (Schrödinger, 2015).

[32] Krissinel E, Henrick K (2007). Inference of macromolecular assemblies from crystalline state. J. Mol. Biol..

