# Supplementary material for: Structure and dynamics of a mycobacterial type VII secretion system
Source: Nature. 2021 May 12;593(7859):445–8. doi: 10.1038/s41586-021-03517-z (PMC8131196; doi:10.1038/s41586-021-03517-z)

nature portfolio

https://doi.org/10.1038/s41586-021-03517-z

Supplementary information

Structure and dynamics of a mycobacterial type VII secretion system

In the format provided by the authors and unedited

Structure and dynamics of a mycobacterial type VII secretion system

Catalin M. Bunduc[1,2,3,4], Dirk Fahrenkamp[1,2,3], Jiri Wald[1.2.3], Roy Ummels[5], Wilbert Bitter[4,5], Edith N.G. Houben[4], Thomas C. Marlovits[1,2,3*]

1. Centre for Structural Systems Biology, Notkestraße 85, 22607 Hamburg, Germany
2. Institute of Structural and Systems Biology, University Medical Center Hamburg-Eppendorf, Notkestraße 85, 22607 Hamburg, Germany
3. German Electron Synchrotron Centre Notkestraße 85, 22607 Hamburg, Germany
4. Section Molecular Microbiology, Amsterdam Institute of Molecular and Life Sciences, Vrije Universiteit Amsterdam, De Boelelaan 1108, 1081 HZ, Amsterdam, The Netherlands
5. Department of Medical Microbiology and Infection Control, Amsterdam Infection & Immunity Institute, Amsterdam UMC, De Boelelaan 1108, 1081 HZ, Amsterdam, The Netherlands

* Corresponding author: marlovits@marlovitslab.org

Supplementary Table 1. Cryo-EM data collection, refinement and validation statistics

	Intact assembly (EMD-12514) (PDB 7NP7)	Intact assembly (EMD-12517) (PDB 7NPR)	Periplasmic assembly (EMD-12518) (PDB 7NPS)	Periplasmic assembly (EMD-12519) No model	Cytosolic bridge (EMD-12520) (PDB 7NPT)	MycP$_5$-free state I (EMD-12521) (PDB 7NPU)	MycP$_5$-free state II (EMD-12522) (PDB 7NPV)	Intact assembly, EccC$_5$ extended (EMD-12523) No model	Intact assembly, EccC$_5$ contracted (EMD-12525) No model
**Data collection and processing**									
Magnification	81000	81000	81000	81000	81000	81000	81000	81000	81000
Voltage (kV)	300	300	300	300	300	300	300	300	300
Electron exposure (e–/Å^2)	59.5	59.5	59.5	59.5	59.5	59.5	59.5	59.5	59.5
Defocus range (μm)	1-2.5	1-2.5	1-2.5	1-2.5	1-2.5	1-2.5	1-2.5	1-2.5	1-2.5
Pixel size (Å)	1.1	1.1	1.1	1.1	1.1	1.1	1.1	1.1	1.1
Symmetry imposed	C1	C3	C1	C3	C1	C1	C1	C1	C1
Initial particle images (no.)	1096816	1096816	1096816	1096816	719037	1096816	1096816	1096816	1096816
Final particle images (no.)	154929	154929	154929	154929	239292	226793	65483	149055	5874
Map resolution (Å)<br>FSC threshold	4.03<br>0.143	3.82<br>0.143	3.81<br>0.143	3.52<br>0.143	3.27<br>0.143	4.48<br>0.143	6.66<br>0.143	4.23<br>0.143	7.58<br>0.143
Map resolution range (Å)	3.7-11.2	3.4-10.2	3.6-6.7	3.3-5.2	3.1-5.6	4-10.4	4.6-19.8	3.7-28	5.1-45
**Refinement**									
Initial model used (PDB code)	4J94, 4KK7, 6SGW, 6SGZ	4J94, 4KK7, 6SGW, 6SGZ	4J94, 4KK7	-	6SGW, 6SGZ	Composite model	Composite model	-	-
Model resolution (Å)<br>FSC threshold				-				-	-
Model resolution range (Å)				-				-	-
Map sharpening *B* factor (Å^2)	+25 to -75	+25 to -75	+25 to -75	-	+25 to -75	+25 to -75	+25 to -75	-	-
Model composition<br>Non-hydrogen atoms<br>Protein residues<br>Ligands	 90176<br>11992<br>-	 90176<br>11992<br>-	 27669<br>3732<br>-	-	 4322<br>552<br>-	 62336<br>8230<br>-	 62336<br>8230<br>-	-	-
B factors (Å^2)<br>Protein (mean)<br>Ligand	 80.7<br>-	 85.8<br>-	 106.40<br>-	-	 47.5<br>-	 93.1<br>-	 106.0<br>-	-	-
R.m.s. deviations<br>Bond lengths (Å)<br>Bond angles (°)	 0.008<br>1.181	 0.008<br>1.216	 0.008<br>1.072	-	 0.008<br>1.247	 0.008<br>1.197	 0.007<br>1.184	-	-
Validation<br>MolProbity score<br>Clashscore<br>Poor rotamers (%)	 0.90<br>1.23<br>0.30	 0.85<br>0.98<br>0.24	 0.91<br>1.03<br>0.00	-	 1.03<br>2.44<br>0.00	 0.85<br>1.26<br>0.37	 0.98<br>2.06<br>0.29	-	-
Ramachandran plot<br>Favored (%)<br>Allowed (%)<br>Disallowed (%)	 97.74<br>2.21<br>0.05	 97.71<br>2.28<br>0.01	 97.48<br>2.52<br>0.00	-	 98.15<br>1.85<br>0.00	 98.06<br>1.83<br>0.11	 98.10<br>1.80<br>0.10	-	-

Supplementary Table 2. Model sequence coverage.

	Single components									Hexameric complex	
				$EccC_5$ without NBD1-3					$MycP_5$ without loop 5		
	$iEccB_5$	$oEccB_5$	$EccC_5$		$iEccD_5$	$oEccD_5$	$EccE_5$	$MycP_5$		Full	Excluding NBD1-3 and loop 5
**Sequence length**	506	506	1391	430	503	503	406	585	475	21609	15513
**Model coverage**	487	485	412	412	493	411	0	451	435	12165	12117
**Percentage**	96.2%	95.8%	29.6%	95.8%	98.0%	81.7%	0.0%	77.1%	91.6%	56.3%	78.1%

Supplementary Table 3. List of primers used in this study.

Nr.	Name	Sequence (5' - 3')
1.	Esx-5 tub 1 Rv	CCCCTTTAAACCGACGCACCTCGGTGGCT
2.	Esx-5 tub 1 Fw	CCAAGGACACTGAGTCCTAA
3.	Esx-5 tub 2 Fw	CTTCTTACTAGTTCCGTCGGCGGGATAGCTTT
4.	Esx-5 tub 2 Rv	CTTCTTCATATGCTCACCCACCGCCGACTGTT
5.	ESX-5 TB strep+substr. FW1	CGAGAAGTAAACTAGTTTCGGCCAAACCGATCAGCTC
6.	ESX-5 TB strep+substr. RV1	CTCGCGTAGGGCTGCAGGTCG
7.	ESX-5 TB strep+substr. FW2	CGACCTGCAGCCCTACGCGAG
8.	ESX-5 TB strep+substr RV2 63	CAATTTGCCCTGTACGTATCTGG

Supplementary Figure 1. Uncropped gels presented in the manuscript

Extended Data Fig. 1b

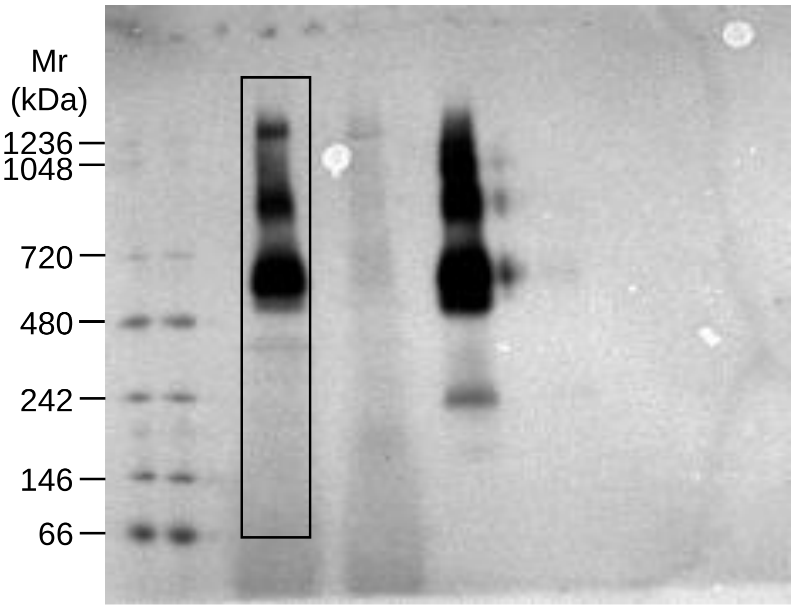

αEccB$_5$

Extended Data Fig. 1b

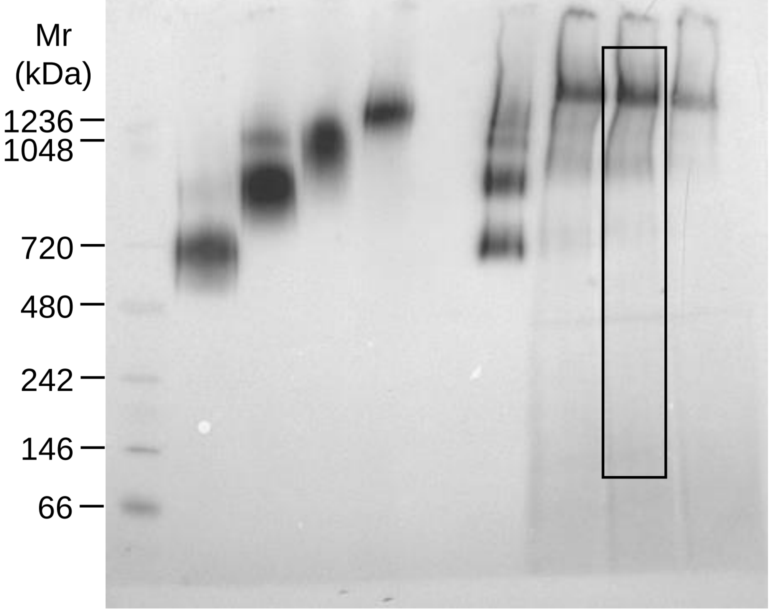

αEccB$_5$

Extended Data Fig. 1c

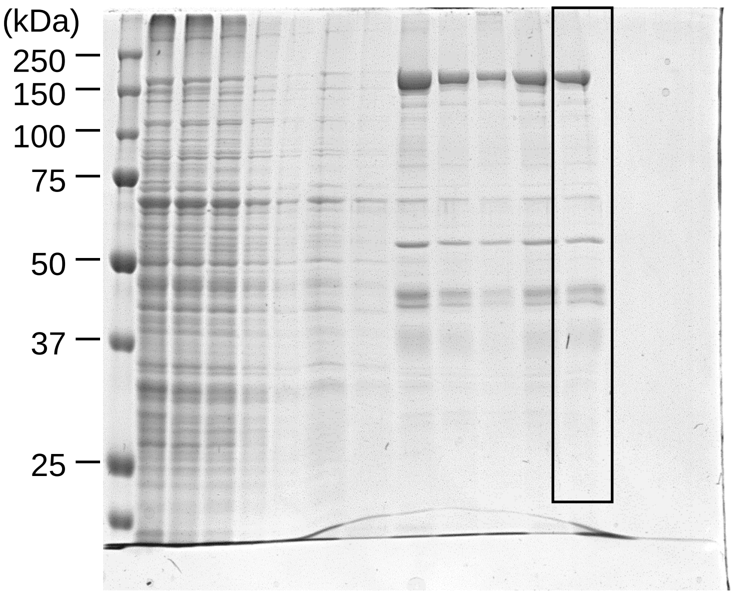

Extended Data Fig. 1d

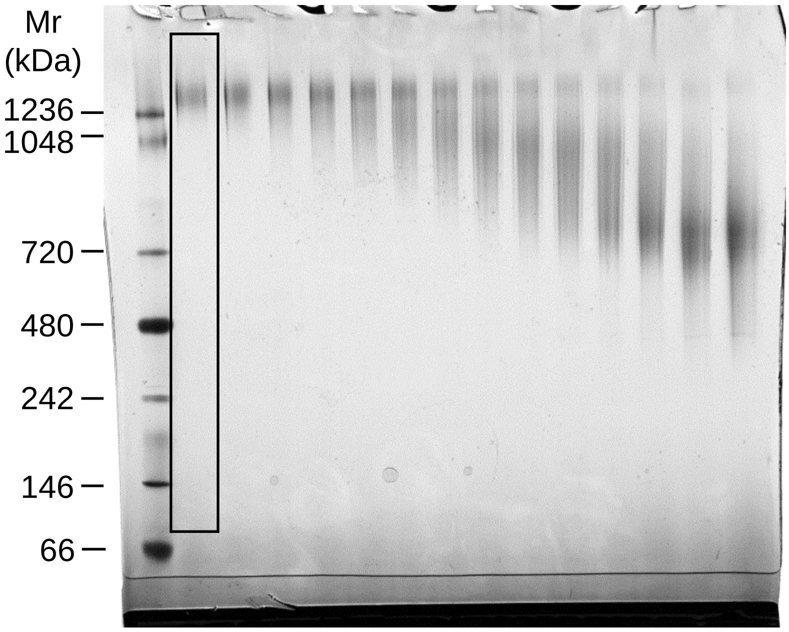

Extended Data Fig. 2a

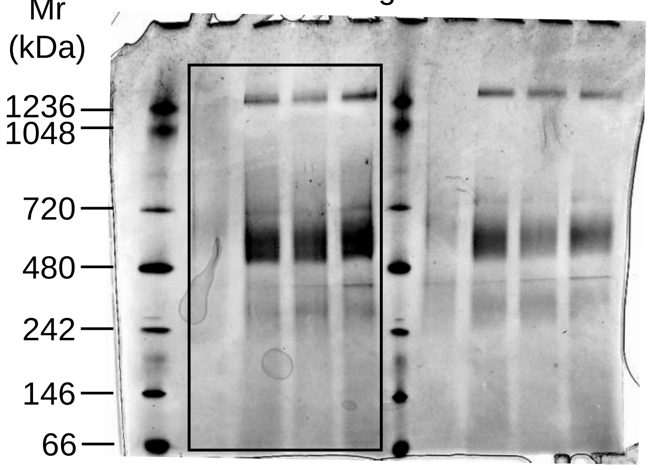

Extended Data Fig. 2b

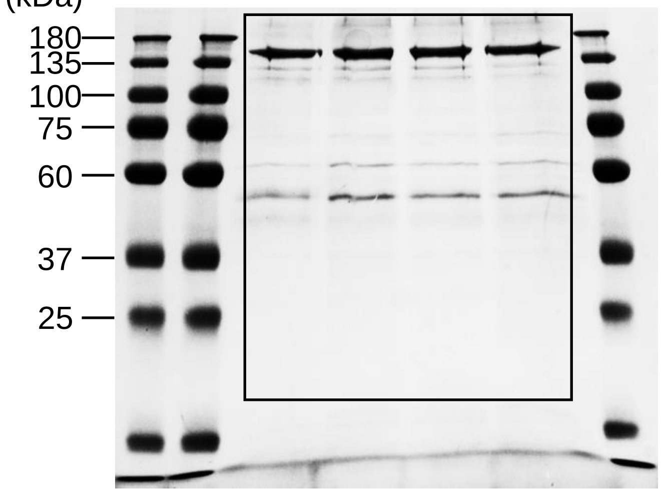

Extended Data Fig. 2c

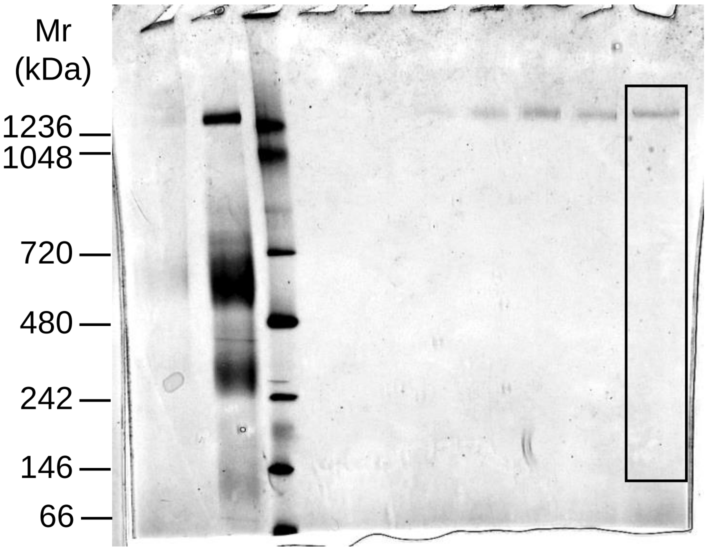

Extended Data Fig. 2d

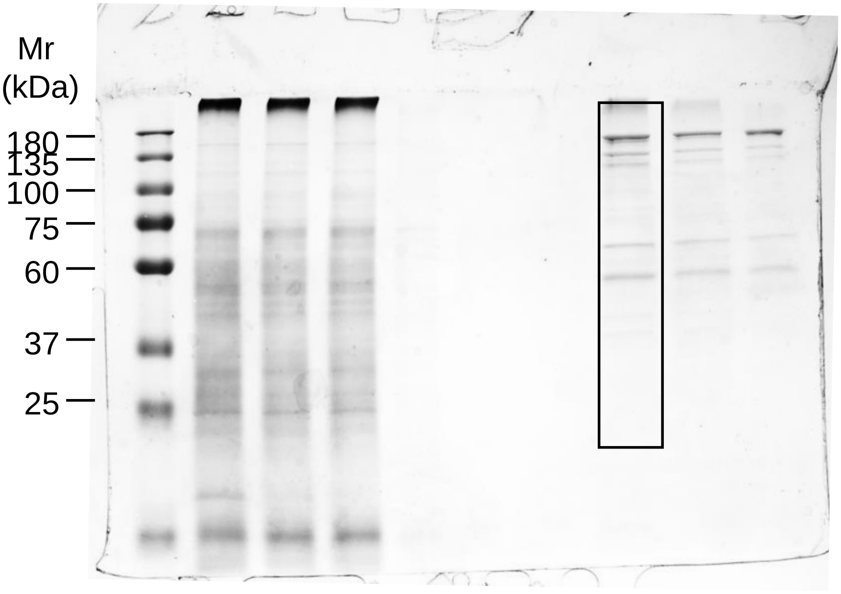

Supplementary Figure 2. Structure based sequence alignment of the five EccB paralogs of *M. tuberculosis* H37Rv. Numbering and secondary structure elements are derived from the $EccB_5$ sequence and structure. Sequences coding for the TMH, domain R1, R2, R3, R4 and two sequences (CD1 and CD2) that code for the central domain (CD) are indicated by black lines. The conserved GIPGAP motif involved in $EccB_5$ dimerization is highlighted. The sequence alignment is produced using ESPript (http://espript.ibcp.fr).

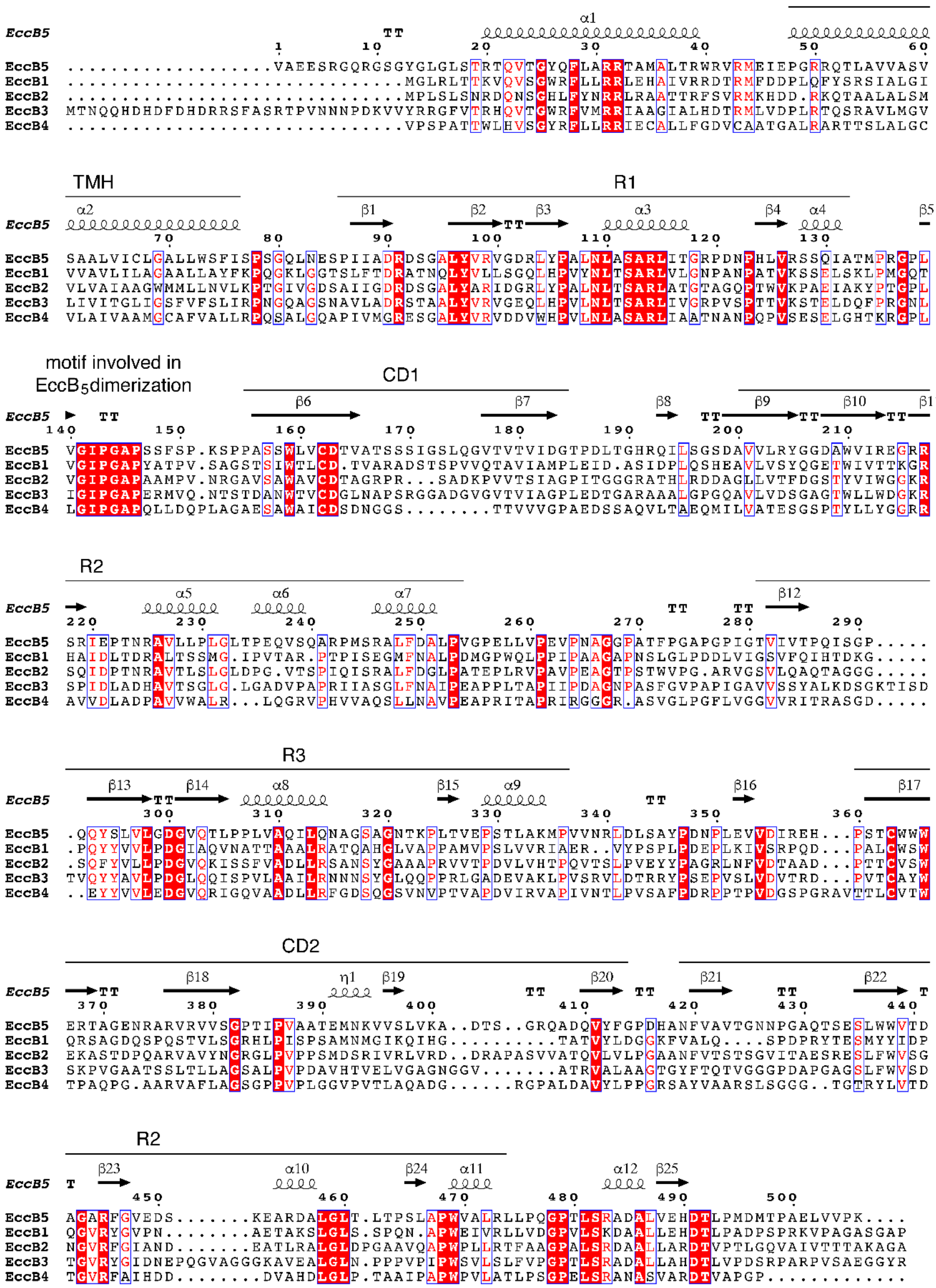

Supplementary Figure 3. Structure based sequence alignment of the five MycP paralogs of *M. tuberculosis* H37Rv. Numbering and secondary structure elements are derived from the $MycP_5$ sequence and structure. The signal protease cleavage site is indicated by a vertical arrow. Sequences coding for the signal sequence, N-terminal extension, loops 1 to 6 and the TMH are indicated by black lines. While loop 1, 2, 3 and 5 have been assigned previously, loop 4 and 6 are newly assigned in this study. The sequence alignment is produced using ESPript (http://espript.ibcp.fr).

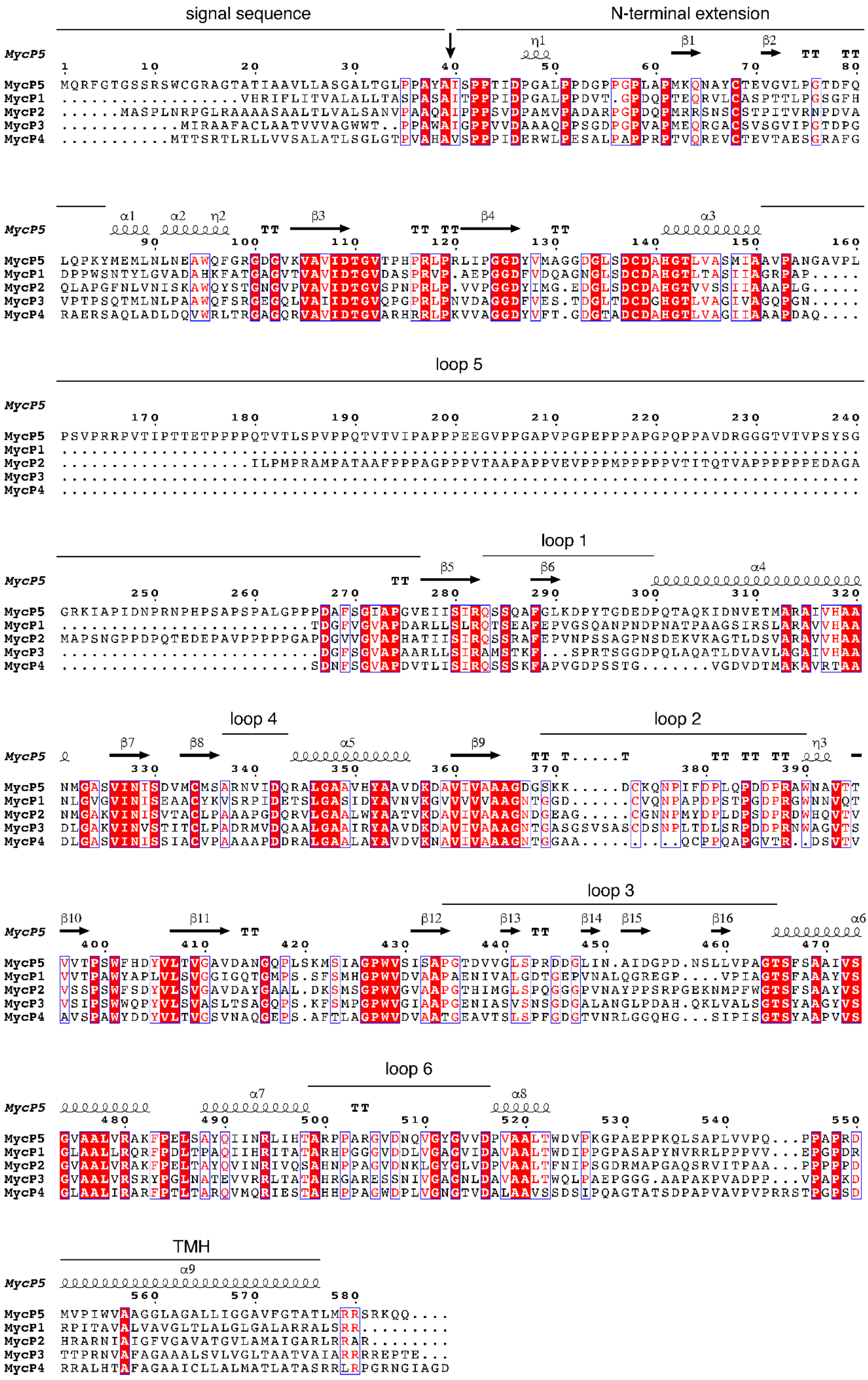

Supplement: Supplementary file 1 — This file contains Supplementary Tables 1-3 and Supplementary Figures 1-3. [file 41586_2021_3517_MOESM1_ESM.pdf]
