# Supplementary material for: Structure and dynamics of a mycobacterial type VII secretion system
Source: Nature. 2021 May 12;593(7859):445–8. doi: 10.1038/s41586-021-03517-z (PMC8131196; doi:10.1038/s41586-021-03517-z)
Supplement: Supplementary file 2 — Reporting Summary. [file 41586_2021_3517_MOESM2_ESM.pdf]

nature research

Corresponding author(s): Thomas C Marlovits

Last updated by author(s): Mar 2, 2021

Reporting Summary

Nature Research wishes to improve the reproducibility of the work that we publish. This form provides structure for consistency and transparency in reporting. For further information on Nature Research policies, see our Editorial Policies and the Editorial Policy Checklist.

Statistics

For all statistical analyses, confirm that the following items are present in the figure legend, table legend, main text, or Methods section.

n/a	Confirmed	
☐	☒	The exact sample size (*n*) for each experimental group/condition, given as a discrete number and unit of measurement
☒	☐	A statement on whether measurements were taken from distinct samples or whether the same sample was measured repeatedly
☒	☐	The statistical test(s) used AND whether they are one- or two-sided<br>*Only common tests should be described solely by name; describe more complex techniques in the Methods section.*
☒	☐	A description of all covariates tested
☒	☐	A description of any assumptions or corrections, such as tests of normality and adjustment for multiple comparisons
☒	☐	A full description of the statistical parameters including central tendency (e.g. means) or other basic estimates (e.g. regression coefficient) AND variation (e.g. standard deviation) or associated estimates of uncertainty (e.g. confidence intervals)
☒	☐	For null hypothesis testing, the test statistic (e.g. *F*, *t*, *r*) with confidence intervals, effect sizes, degrees of freedom and *P* value noted<br>*Give P values as exact values whenever suitable.*
☒	☐	For Bayesian analysis, information on the choice of priors and Markov chain Monte Carlo settings
☒	☐	For hierarchical and complex designs, identification of the appropriate level for tests and full reporting of outcomes
☒	☐	Estimates of effect sizes (e.g. Cohen's *d*, Pearson's *r*), indicating how they were calculated

Our web collection on statistics for biologists contains articles on many of the points above.

Software and code

Policy information about availability of computer code

Data collection	CryoEM: Thermo Fisher EPU V1.11 and V2.4; Negative stain EM: Thermo Fisher TIA V4.1.5;<br>Western blotting: ChemoStar Touch (Intas Science Imaging Instruments GmbH, v. 0.5.65).
Data analysis	MotionCor2 v.1.2.1 and v.1.3, CTFfind v.4.1.13, crYOLO v.1.4, Relion 3.1-beta, Chimera v.1.13.1 and 1.14, ChimeraX v.1 and v.1.1, Pymol v.2.40, Phyre2 (unversioned), Phenix v.1.18.2, Density modification (unversioned) and EMRinger (unversioned) both within the Phenix suite, DeepEMhancer (unversioned), ISOLDE v.1.0b5, MolProbity (unversioned), Phenix.real_space_refine v.1.18-6831, PISA (unversioned).

For manuscripts utilizing custom algorithms or software that are central to the research but not yet described in published literature, software must be made available to editors and reviewers. We strongly encourage code deposition in a community repository (e.g. GitHub). See the Nature Research guidelines for submitting code & software for further information.

Data

Policy information about availability of data

All manuscripts must include a data availability statement. This statement should provide the following information, where applicable:

- Accession codes, unique identifiers, or web links for publicly available datasets
- A list of figures that have associated raw data
- A description of any restrictions on data availability

Cryo-EM maps have been deposited in the Electron Microscopy Database under accession codes EMD-12514 (full complex in C1), EMD-12517 (full complex in C3), EMD-12518 (periplasmic map in C1), EMD-12519 (periplasmic map in C3), EMD-12520 (cytosolic bridge), EMD-12521 (MycP5-free map 1), EMD-12522 (MycP5-free map 2), EMD-12523 (EccC5 extended state) and EMD-12525 (EccC5 contracted state). The composite model settled in the C1 and C3 full maps, periplasm in C1, cytosolic bridge, MycP5-free map 1 and MycP5-free map 2 have been deposited in the Protein Data Bank under PDB accession codes 7NP7, 7NPR, 7NPS, 7NPT, 7NPU and 7NPV respectively. All other data is available from the corresponding author upon reasonable request.

Field-specific reporting

Please select the one below that is the best fit for your research. If you are not sure, read the appropriate sections before making your selection.

[X] Life sciences [ ] Behavioural & social sciences [ ] Ecological, evolutionary & environmental sciences

For a reference copy of the document with all sections, see nature.com/documents/nr-reporting-summary-flat.pdf

Life sciences study design

All studies must disclose on these points even when the disclosure is negative.

Sample size	Sample sizes were chosen as a maximum possible while considering practical limitations for data collection and subsequent data processing. The size of the final particle set was determined by the ability to reach resolutions better than 4 Å in 3D reconstructions.
Data exclusions	Data was excluded during cryo-EM data processing by removing 2D and 3D classes that did not posses high-resolution features, a standard method for cryo-EM high resolution structural determination.
Replication	All experiments have been successfully replicated. Solubilized membranes have been analyzed with BN-PAGE and EccB5 immunostaining three times (ED Fig. 1B). Purifications +/- nucleotides (ED Fig. 1f, g) was replicated three times. Purification without nucleotides with EM assessment has been performed three times (ED Fig. 1c, d, e) and purification with nucleotides together with cryo-EM data collection has been performed two times (ED Fig. 1 h, i, j).
Randomization	Particles/images were randomly partitioned for resolution and quality assesment.
Blinding	Blinding during data collection and analysis is not a commonly applied procedure in cryo-EM.

Behavioural & social sciences study design

All studies must disclose on these points even when the disclosure is negative.

Study description	Not relevant to this study
Research sample	Not relevant to this study
Sampling strategy	Not relevant to this study
Data collection	Not relevant to this study
Timing	Not relevant to this study
Data exclusions	Not relevant to this study
Non-participation	Not relevant to this study
Randomization	Not relevant to this study

Ecological, evolutionary & environmental sciences study design

All studies must disclose on these points even when the disclosure is negative.

Study description	Not relevant to this study
Research sample	Not relevant to this study
Sampling strategy	Not relevant to this study
Data collection	Not relevant to this study
Timing and spatial scale	Not relevant to this study
Data exclusions	Not relevant to this study
Reproducibility	Not relevant to this study
Randomization	Not relevant to this study

Blinding: Not relevant to this study

Did the study involve field work? ☐ Yes ☒ No

Reporting for specific materials, systems and methods

We require information from authors about some types of materials, experimental systems and methods used in many studies. Here, indicate whether each material, system or method listed is relevant to your study. If you are not sure if a list item applies to your research, read the appropriate section before selecting a response.

Materials & experimental systems

n/a	Involved in the study
☐	☒ Antibodies
☒	☐ Eukaryotic cell lines
☒	☐ Palaeontology and archaeology
☒	☐ Animals and other organisms
☒	☐ Human research participants
☒	☐ Clinical data
☒	☐ Dual use research of concern

Methods

n/a	Involved in the study
☒	☐ ChIP-seq
☒	☐ Flow cytometry
☒	☐ MRI-based neuroimaging

Antibodies

Antibodies used: anti EccB5 antibody

Validation: The anti EccB5 antibody was raised against the synthetic peptide CLPMDMSPAELVVPK by Innovagen (Lund, Sweden) and has been validated in doi:10.1111/j.1365-2958.2012.08206.x. It was used as a 1:5000 dilution.
